# Empiric treatment against invasive fungal diseases in febrile neutropenic patients: a systematic review and network meta-analysis

**DOI:** 10.1186/s12879-017-2263-6

**Published:** 2017-02-20

**Authors:** Ken Chen, Qi Wang, Roy A. Pleasants, Long Ge, Wei Liu, Kangning Peng, Suodi Zhai

**Affiliations:** 1 Department of Pharmacy, Peking University Third Hospital, Beijing, 100191 China; 2 Evidence Based Medicine Center, School of Basic Medical Sciences, Lanzhou University, Lanzhou, 730000 China; 3 Duke University Division of Pulmonary, Allergy, and Critical Care Medicine, Duke University School of Medicine, Durham, NC 27705 USA; 4 School of Basic Medical Sciences, Peking University Health Science Center, Beijing, 100191 China

**Keywords:** Antifungals, Febrile neutropenia, Empiric therapy, Systematic review, Network meta-analysis

## Abstract

**Background:**

The most optimal antifungal agent for empiric treatment of invasive fungal diseases (IFDs) in febrile neutropenia is controversial. Our objective was evaluate the relative efficacy of antifungals for all-cause mortality, fungal infection-related mortality and treatment response in this population.

**Methods:**

Pubmed, Embase and Cochrane Library were searched to identify randomized controlled trials (RCTs). Two reviewers performed the quality assessment and extracted data independently. Pairwise meta-analysis and network meta-analysis were conducted to compare the antifungals.

**Results:**

Seventeen RCTs involving 4583 patients were included. Risk of bias of included studies was moderate. Pairwise meta-analysis indicated the treatment response rate of itraconazole was significantly better than conventional amphotericin B (RR = 1.33, 95%CI 1.10–1.61). Network meta-analysis showed that amphotericin B lipid complex, conventional amphotericin B, liposomal amphotericin B, itraconazole and voriconazole had a significantly lower rate of fungal infection-related mortality than no antifungal treatment. Other differences in outcomes among antifungals were not statistically significant. From the rank probability plot, caspofungin appeared to be the most effective agent for all-cause mortality and fungal infection-related mortality, whereas micafungin tended to be superior for treatment response. The results were stable after excluding RCTs with high risk of bias, whereas micafungin had the lowest fungal infection-related mortality.

**Conclusions:**

Our results highlighted the necessity of empiric antifungal treatment and indicates that echinocandins appeared to be the most effective agents for empiric treatment of febrile neutropenic patients based on mortality and treatment response. However, more studies are needed to determine the best antifungal agent for empiric treatment. Our systematic review has been prospectively registered in PROSPERO and the registration number was CRD42015026629.

**Electronic supplementary material:**

The online version of this article (doi:10.1186/s12879-017-2263-6) contains supplementary material, which is available to authorized users.

## Background

Invasive fungal diseases (IFDs) are crucial causes of morbidity and mortality among febrile neutropenic (FN) patients after intensive chemotherapy or hematopoietic stem cell transplantation (HSCT) as well as in other immunocompromised populations [1–3]. Mortality rates exceed 30% in patients diagnosed with IFDs [4–6]. In the past several decades, increasing numbers of susceptible hosts, introduction of newer modalities for HSCT, and current broad-spectrum antimicrobial therapy strategies have contributed to the high frequency of IFDs [7].

Diagnosis of IFDs is categorized as proven, probable or possible [8]. Proven IFD is defined as demonstration of fungal elements in infected tissue for most conditions irrespective of host factors or clinical features. Cases of probable IFDs require a host factor, clinical features, and mycological evidence. Possible IFDs include cases with appropriate host factors and sufficient clinical evidence but no mycological support. These definitions have been adopted by most practice guidelines for IFDs. The most commonly identified fungal species associated with IFD are Candida species, Aspergillus, Cryptococcus and Pneumocystis [9].

In spite of the significant improvement in diagnostic tests, accurate diagnosis of IFDs remains challenging, particularly in patients with hematologic malignancies. Patients with hematologic malignancies often present with non-specific signs and symptoms that have developed late in the course of infection. Empiric antifungal treatment is frequently prescribed, either on initial presentation, or after other potential causative bacteria have been treated. A previous systematic review in 2008 showed that the addition of empiric antifungal therapy in patients with FN significantly improved IFDs outcomes compared to no antifungal [10].

Several organizations have published guidelines with treatment recommendations for FN, as well as recommendations on organism-specific treatment of IFDs including the Infectious Diseases Society of America (IDSA) [11, 12], European Conference on Infections in Leukemia (ECIL) [13]. The 2016 IDSA published guidelines stratify FN patients based on presumed duration and severity of neutropenia, as well as other co-morbidities [12]. Empiric antifungal therapy is recommended in high-risk patients for IFD who have persistent fever after 4–7 days of broad-spectrum antibacterials and no identified infection source [13].

Despite a significant number of published guidelines regarding the treatment of IFD, recommendations fail to reach a consensus on preferred antifungal therapy in patients with FN. Further evaluation of available literature is indicated to provide consistent recommendations for optimizing antifungal therapy for the treatment of IFDs.

In this article, we aimed to evaluate the relative effectiveness of antifungal agents as empiric therapy in FN patients for all-cause mortality, fungal infection-related mortality, and treatment response via pairwise and network meta-analysis.

## Methods

### Registry

The review was prospectively registered on the Centre for Reviews and Dissemination, University of York. The registration number was CRD42015026629.

### Search strategy

Pubmed, Embase and Cochrane Library were searched until May 21st, 2016. References of retrieved articles and relative systematic reviews were also identified. The search terms were the combination of subject terms and free terms. Names of antifungal agents, neutropenia and fever were combined as search terms. A sample search strategy of Medline was provided in Additional file 1.

### Selection criteria

Randomized controlled trials (RCTs) in English or Chinese on use of systemic, empiric antifungal agents were eligible for inclusion. Empiric antifungal treatment was defined as antifungal treatment for FN patients with poor response to ≥3 days of broad-spectrum antibacterial therapy and without radiological or microbiological evidence of IFDs. Studies of flucytosine, ketoconazole, miconazole and nystatin as well as combination therapies were excluded from analysis. Dosage of antifungal agents was consistent with US Food and Drug Administration (FDA)-approved package inserts, respectively.

### Data extraction and outcomes

The following data was extracted from identified studies: study design, patient characteristics, intervention and comparison, sample size, etc. The primary outcome was all-cause mortality; secondary outcomes included fungal infection-related mortality and treatment response. Given the known variation in the definitions of treatment response, we chose to use the criteria from the majority of included studies to minimize heterogeneity.

### Quality appraisal

The Cochrane risk of bias tool was applied to evaluate the quality of included RCTs [14]. Because we judged outcomes were not likely to be influenced by lack of blinding of participants and personnel, the item was rated as “low risk of bias” for all studies. Additionally, since all-cause mortality was unlikely biased by lack of blinding assessment, the judgment for blinding assessment of outcomes was only used for fungal infection-related mortality and treatment response.

### Geometry of the network

A network plot was drawn to describe and present the geometry of the treatment network of comparisons across trials to ensure if a network meta-analysis was feasible. Trials were excluded if the trials were not connected by treatments. Network geometry used nodes to represent different interventions and edges to represent the head-to-head comparisons [15].

### Statistical analysis

Pairwise meta-analysis for each head-to-head comparison was performed using RevMan 5.1 (Cochran IMS) respectively. The Mantel–Heanzel method was used as the statistical model to calculate the risk ratio (RR) and 95% confidence intervals (CI) for pooled outcomes. The Cochran Q *χ*2 test and I^2^ statistic were used to assess heterogeneity among studies. *P* < 0.1 was considered significant because of the low statistical power of the *χ*2 test for heterogeneity. The random-effect model was always used for pairwise meta-analysis with regard to potential heterogeneity among studies.

Network meta-analysis (NMA) was conducted using ADDIS version 1.16.6 software. The pooled estimation and the probability of the best treatment were obtained using the Markov Chains Monte Carlo method. Using a full Bayesian evidence network, all indirect comparisons were taken into account to arrive at a single, integrated, estimate of the effect of all included arms based on all studies. A consistency model was used to draw conclusions about the relative effect of the treatments. The rank probability plot by the NMA was used to determine which empiric antifungal treatment was the best for each outcome. A node-splitting analysis was performed to assess inconsistency between direct and indirect evidence on a specific node in NMA. It was deemed significant when P was less than 0.05. Convergence was assessed using the Brooks-Gelman-Rubin method [16]. Four Markov Chains were run simultaneously with different arbitrarily chosen initial values. Subgroup analysis was conducted based on proportion of allogenic hematologic stem cell transplantation (allo-HSCT) and ages, while sensitivity analysis was conducted based on methodological quality of included studies.

## Results

### Characteristics and quality appraisal of included studies

One thousand four hundred forty-three references were initially identified. After selection, 17 studies met our inclusion criteria (Additional files 1 and 2) [17–33]. References of four systematic reviews were retrieved and no additional studies were added into our systematic review [34–37]. There were 4583 patients in the included studies that were conducted in different countries as follows: United States (*n* = 2) [28, 32], Italy (*n* = 2) [18, 27], Germany (*n* = 3) [19, 25, 26], Japan (*n* = 1) [23], China (*n* = 1) [31], India (*n* = 1) [20], Korea (*n* = 1) [33] and multi-countries (*n* = 6) [17, 21, 22, 24, 29, 30]. Differences across studies in the formulation administered were observed for fluconazole, itraconazole, and voriconazole.

**Fig. 1:**
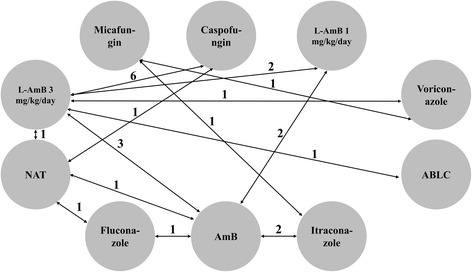
A schematic of the network of evidence used in network meta-analysis. ABLC: Amphotericin B lipid complex; AmB: Conventional amphotericin B; L-AmB: Liposomal amphotericin B; NAT: No antifungal treatment

Characteristics of included studies are presented in Table 1. Raw data is presented in Additional file 1. Most patients suffered from hematologic malignancy and were neutropenic after intensive chemotherapy or HSCT. Timing of empiric antifungal treatment varied from 3 to 7 days after initiating broad-spectrum antibacterial therapy. The most common pathogens that were detected after initiation of antifungal therapy were *Candida* and *Aspergillus*. Treatment response was principally defined as absence of IFDs, completion of therapy, fever resolution, and survival during the follow-up period. Antifungal therapy was usually continued until recovery of neutropenia. Caselle et al. conducted a stratified randomization based on risk of IFDs where no antifungal treatment (NAT) was given to the low risk group, we separated it into two studies [18]. Daily dosage of 1 and 3 mg/kg/day of liposomal amphotericin B (L-AmB) was separated as two arms regarding variance between the two dosages. From the included studies, a network of evidence for eight alternative empiric antifungal agents and NAT (ten arms) and 14 head-to-head comparisons was constructed (Fig. 1). RCTs studying L-AmB 3 mg/kg/day, caspofungin and conventional amphotericin B (AmB) were relatively abundant (three studies that included itraconazole, two for voriconazole, two for fluconazole, ten for L-AmB, seven for AmB, one for amphotericin B lipid complex (ABLC), five for caspofungin, and two for micafungin.

**Table 1:** Characteristic of included studies

References	Study design, country	Patient population	Allo-HSCT percentage	Interventions and sample sizes (n)			Outcomes
				A	B	C	
Boogaerts 2001 [17]	Open, multicenter, randomized, controlled clinical trial, multi-countries (North America, Europe and Oceania)	Age ≥ 18, hematologic cancer with intensive myelosuppressive cytotoxic therapy or auto-HSCT support.	0	Itraconazole 200 mg iv. q12h for the first 48 h, followed by 200 mg iv. qd from days 3 to 14. From day 15, 400 mg/d po (*n* = 179).	C-AmB 0.7–1.0 mg/kg iv. qd (*n* = 181)	/	①②③
Caselli 2012 [18]	Multicentre, randomized, controlled trial, Italy	Age ≤ 18, with at least one of the following features: AML, early relapse of ALL, ongoing auto-HSCT with bone marrow as the source of the stem cells, a neutropenic score ≥5.	0	L-AmB 3 mg/kg iv. qd (*n* = 25)	Caspofungin at a LD of 70 mg/m^2^ iv. on day 1, then 50 mg/m^2^ iv. qd (*n* = 31)	/	③
		Age ≤ 18, without other features mentioned above.	0	L-AmB 3 mg/kg iv. qd (*n* = 15)	Caspofungin at a LD of 70 mg/m^2^ iv. on day 1, then 50 mg/m^2^ iv. qd (*n* = 17)	NAT(*n* = 16)	
Groll 2010 [19]	Open, prospective, randomized multicenter phase II trial, Germany	Age ≥ 18, with allo-HSCT and immunosuppression with cyclosporine.	100%	Caspofungin at a LD of 70 mg iv. on day 1, then 50 mg iv. qd (*n* = 18)	L-AmB 3 mg/kg iv. qd (*n* = 20)	/	①②③
Jadhav 2012 [20]	Randomized, multicenter trial, India	Age > 2 and < 60, with chemotherapy or BMT.	NR	L-AmB 3 mg/kg iv. qd (*n* = 23)	C-AmB 1 mg/kg iv. qd (*n* = 20)	L-AmB 1 mg/kg iv. qd (*n* = 22)	①②③
Jeong 2016 [33]	Randomized, controlled, prospective, multicenter study, Korea	Age ≥ 18, with intensive anticancer chemotherapy for acute leukemia, highly aggressive lymphoma, or other hematological malignancies.	0	Micafungin 100 mg iv. qd (*n* = 73)	Itraconazole200 mg iv. bid on Day 1 and 2, then 200 mg iv. qd (*n* = 75)	/	①③
Maertens 2010 [21]	Prospective, randomized, double-blind study, multi-countries (North America and Europe)	Age > 2 and < 17, with allo-HSCT or chemotherapy for a relapse of AML or ALL.	40.9%	Caspofungin 70 mg/m^2^ LD iv. on Day 1, then 50 mg/m^2^ iv. qd (maximum 70 mg/d) plus placebo corresponding to L-AmB (*n* = 15)	L-AmB 3 mg/kg iv. qd plus placebo corresponding to caspofungin (*n* = 7)	/	①③
		Age > 2 and < 17, without allo-HSCT or chemotherapy for a relapse of AML or ALL.	0	Casfofungin 70 mg/m^2^ LD iv. on Day 1, then 50 mg/m^2^ qd (maximum 70 mg/d) plus placebo corresponding to L-AmB (*n* = 41)	L-AmB 3 mg/kg iv. qd plus placebo corresponding to Caspofungin (*n* = 18)	/	
Meunier 1989 [22]	Multicenter Randomized trial, multi-countries (Europe)	Age > 15.	NR	AmB 1.2 mg/kg iv. qod or 0.6 mg/kg iv. qd (*n* = 57)	NAT (*n* = 51)	/	①②③
		Age ≤ 15.	NR	AmB 1.2 mg/kg iv. qod or 0.6 mg/kg iv. qd (*n* = 11)	NAT (*n* = 13)	/	
Oyake 2015 [23]	open-label, randomized, multicenter, comparative trial, Japan	Age ≥ 16.	2.0%	Micafungin 150 mg iv. qd (*n* = 49)	Voriconazole at a LD of 6 mg/kg iv. bid on day 1 followed by 4 mg/kg iv. bid (*n* = 45)	/	①②③
Prentice 1997 [24]	Prospective, open-label, randomized, multicenter trial, multi-countries (Europe)	Adult patients.	NR	AmB 1 mg/kg iv. qd (n = 39)	L-AmB 3 mg/kg iv. qd (n = 47)	L-AmB 1 mg/kg iv. qd (*n* = 47)	③
		Pediatric patients.	NR	AmB 1 mg/kg iv. qd (*n* = 61)	L-AmB 3 mg/kg iv. qd (*n* = 71)	L-AmB 1 mg/kg iv. qd (*n* = 70)	
Schiel 2006 [25]	Randomized, controlled multi-center trial, Germany	Age ≥ 18, with high grade hematological disorders.	0	NAT (*n* = 54)	Fluconazole 800 mg iv. at day 1, followed by 400 mg iv qd (*n* = 56)	/	①③
Schuler 2007 [26]	Open, randomized, multicenter, parallel-group trial, Germany	Age ≥ 18, with haematological malignancy and allo-HSCT.	100%	Itraconazole at a LD of 200 mg iv. q12h for 2 days, 200 mg iv. qd for 12 days, then solution 200 mg po. q12h (*n* = 26)	AmB 0.7–1 mg/kg iv. qd(*n* = 24)	/	①②③
		Age ≥ 18, with haematological malignancy and without allo-HSCT.	0	Itraconazole at a LD of 200 mg iv. q12h for 2 days, 200 mg iv. qd for 12 days, then solution 200 mg po. q12h (*n* = 55)	AmB 0.7–1 mg/kg iv. qd(*n* = 57)	/	
Viscoli 1996 [27]	Prospective, randomised, multicentre, open-label study, Italy	With cancer (including autologous or allogeneic BMT for a neoplastic disease).	41.1%	Fluconazole 6 mg/kg iv. qd (maximum 400 mg/day) (*n* = 56)	AmB 0.8 mg/kg iv. qd (*n* = 56)	/	①②③
Walsh 1999 [28]	Randomized, double-blind, multicenter trial, United states	Age > 2 and < 80, with chemotherapy for leukemia, lymphoma, or other cancers, or with BMT or peripheral HSCT.	NR	L-AmB 3 mg/kg iv. qd (*n* = 343)	AmB 0.6 mg/kg iv. qd (*n* = 344)	/	①②③
Walsh 2002 [29]	Open-label, prospective, randomized, multicenter, international comparative trial, multi-countries (Europe and North America)	Age ≥ 12, with allo-HSCT or chemotherapy for relapsed leukemia.	54.6%	Voriconazole at a LD of 6 mg/kg iv. q12h on day 1 followed by 3 mg/kg iv. q12h or 200 mg po. q12h after at least 3 days of intravenous therapy (*n* = 143)	L-AmB 3 mg/kg iv. qd (*n* = 141)	/	①②③
		Age ≥ 12, without allo-HSCT or chemotherapy for relapsed leukemia.	0	Voriconazole at a LD of 6 mg/kg iv. q12h on day 1 followed by 3 mg/kg iv. q12h or 200 mg po. q12h after at least 3 days of intravenous therapy (*n* = 272)	L-AmB 3 mg/kg iv. qd (*n* = 281)	/	
Walsh 2004 [30]	Prospective, double-blind study, multi-countries (North America, South America, Europe, Asia and Oceania)	Age ≥ 16, with allo-HSCT or chemotherapy for relapsed leukemia.	28.0%	Caspofungin 70 mg iv. LD on Day 1, then 50 mg iv. qd plus placebo corresponding to L-AmB	L-AmB 3 mg/kg iv. qd plus placebo corresponding to caspofungin	/	①③
		Age ≥ 16, without allo-HSCT or chemotherapy for relapsed leukemia.	0	Caspofungin 70 mg iv. LD on Day 1, then 50 mg qd plus placebo corresponding to L-AmB	L-AmB 3 mg/kg iv. qd plus placebo corresponding to caspofungin	/	
Wang 2007 [31]	Open, randomized, controlled trial, China	With immunosuppression, long term use of glucocorticoid or neutropenia after radiotherapy and chemotherapy.	41.7%	Caspofungin at a LD of 70 mg iv. on day 1, then 50 mg iv. qd (*n* = 32)	L-AmB 3 mg/kg iv. qd (*n* = 28)	/	①③
Wingard 2000 [32]	Randomized, double-blind comparative Trial, United States	Age > 2.	15.3%	L-AmB 3 mg/kg iv. qd (*n* = 85)	ABLC 5 mg/kg iv. qd (*n* = 78)	/	①②③

①All-cause mortality; ②Fungal infection-related mortality; ③Treatment response

*ABLC* Amphotericin B lipid complex, *ALL* Acute lymphocytic leukemia, *Allo-HSCT* Allogeneic hematopoietic stem cell transplantation, *AML* Acute myelocytic leukemia, *Auto-HSCT* Autologous hematopoietic stem cell transplantation, *BMT* Bone marrow transplantation, *AmB* Conventional amphotericin B, *L-AmB* Liposomal amphotericin B, *LD* Loading dose, *NAT* No antifungal treatment, *NR* Not reported

Quality appraisal of included studies was presented in Additional file 2 according to the Cochrane Risk of Bias Tool. Of the included 17 studies, four reached a full score by the risk of bias tool [21, 28–30]. Selection bias showed unclear risk in nine studies [18–20, 22, 23, 25, 26, 31, 32], while the item of blinding of outcome assessment was judged high and unclear risk for four and four studies, respectively [17–20, 24, 27, 31, 33]. Outcome data completion of three studies were considered high risk of bias due to lack of intention-to-treatment (ITT) [17, 18, 22].

### Pairwise meta-analysis

Estimates from pairwise meta-analysis of the relative effectiveness of all agents are presented in Table 2. Forest plots are presented in Additional file 2. Of 14 head-to-head comparisons, only five had pooled data of fungal infection-related mortality, along with a zero rate for either agent treatment in four comparisons. According to the results of head-to-head comparisons, itraconazole was significantly better than AmB for treatment response rate (RR = 1.33, 95%CI 1.10–1.61). Apart from this, there were no statistical differences among all agents by pairwise meta-analysis.

**Table 2:** Estimates from pairwise meta-analysis of the relative efficacy

Comparison, No. of studies	All-cause mortality			Fungal infection-related mortality			Treatment response		
	RR (95% CI)	I^2^	N	RR (95% CI)	I^2^	N	RR (95% CI)	I^2^	N
Itraconazole vs AmB, 2 RCTs	0.88 (0.57, 1.36)	0	522	0.61 (0.14, 2.56)	0	522	1.33 (1.10, 1.61)	0	522
L-AmB^a^ vs Caspofungin, 6 RCTs	1.43 (0.98, 2.08)	0	1274	/	/	/	0.97 (0.87, 1.08)	0	1362
L-AmB^a^ vs NAT, 1 RCT	/	/	/	/	/	/	0.91 (0.67, 1.25)	/	31
Caspofungin vs NAT, 1 RCT	/	/	/	/	/	/	1.08 (0.86, 1.34)	/	33
AmB vs Fluconazole, 1 RCT	0.67 (0.12, 3.84)	/	112	/	/	/	0.88 (0.69, 1.12)	/	112
AmB vs NAT, 1 RCT	0.74 (0.36, 1.51)	/	132	0.10 (0.01, 1.91)	/	132	1.30 (0.98, 1.72)	/	132
Micafungin vs Voriconazole, 1 RCT	2.76 (0.12, 66.07)	/	94	/	/	/	1.05 (0.77, 1.42)	/	94
NAT vs Fluconazole, 1 RCT	0.35 (0.01, 8.30)	/	110	/	/	/	0.89 (0.65, 1.22)	/	110
Voriconazole vs L-AmB^a^, 1 RCT	1.34 (0.81, 2.22)	/	837	0.51 (0.05, 5.59)	/	837	0.85 (0.69, 1.06)	/	837
Micafungin vs Itraconazole, 1 RCT	0.77 (0.28, 2.11)	/	148	/	/	/	1.12 (0.87, 1.46)	/	148
L-AmB^a^ vs AmB, 3 RCTs	0.73 (0.46, 1.17)	0	730	0.36 (0.12, 1.13)	/	687	1.09 (0.91, 1.31)	0.36	948
L-AmB^a^ vs L-AmB^b^, 2 RCTs	0.96 (0.22, 4.24)	/	45	/	/	/	1.03 (0.82, 1.29)	0.14	280
AmB vs L-AmB^b^, 2 RCTs	0.73 (0.14, 3.95)	/	42	/	/	/	0.85 (0.68, 1.06)	0	259
L-AmB^a^ vs ABLC, 1 RCT	0.42 (0.15, 1.15)	/	163	0.31 (0.03, 2.88)	/	163	1.20 (0.80, 1.80)	/	163

*RR* Risk ratio, *CI* Confidence interval, *AmB* Conventional amphotericin B, *RCT* Randomized controlled trial, *L-AmB* Liposomal amphotericin B, *NAT* No antifungal treatment, *ABLC* Amphotericin B lipid complex

^a^3 mg/kg/day; ^b^1 mg/kg/day

### Network meta-analysis

#### All-cause mortality

**Fig. 2:**
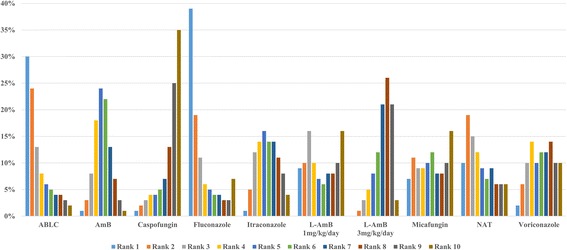
Rank probability plot of antifungals for all-cause mortality. ABLC: Amphotericin B lipid complex; AmB: Conventional amphotericin B; L-AmB: Liposomal amphotericin B; NAT: No antifungal treatment. Rank 1 is worst, rank 10 is best

Fifteen studies (ten arms, 4225 patients) were included for all-cause mortality (Additional file 2) [17, 19–23, 25–33]. According to the results of NMA, there were no statistical differences among all agents (Table 3). From the rank probability plot, caspofungin appeared to have the lowest rate of all-cause mortality (Fig. 2).

**Table 3:** The network meta-analysis results (presented as odds ratio) for all-cause mortality

ABLC	AmB	Caspofungin	Fluconazole	Itraconazole	L-AmB^a^	L-AmB^b^	Micafungin	NAT	Voriconazole
2.13 (0.25, 15.53)									
4.00 (0.36, 30.31)	2.00 (0.29, 9.16)								
0.82 (0.03, 13.01)	0.44 (0.04, 3.16)	0.24 (0.01, 2.79)							
2.23 (0.19, 18.54)	1.06 (0.32, 2.75)	0.55 (0.08, 4.32)	2.38 (0.27, 32.66)						
2.30 (0.13, 30.41)	1.00 (0.13, 8.32)	0.53 (0.05, 6.77)	2.51 (0.14, 58.87)	0.97 (0.10, 10.44)					
2.92 (0.51, 17.19)	1.44 (0.43, 4.65)	0.72 (0.24, 3.10)	3.17 (0.38, 54.22)	1.34 (0.31, 7.65)	1.45 (0.18, 10.99)				
2.28 (0.10, 27.64)	1.10 (0.11, 5.97)	0.53 (0.04, 5.76)	2.53 (0.16, 43.72)	1.03 (0.17, 4.87)	1.14 (0.05, 15.78)	0.72 (0.07, 5.08)			
1.57 (0.13, 26.34)	0.79 (0.18, 4.60)	0.41 (0.05, 5.77)	1.93 (0.13, 34.81)	0.74 (0.15, 6.87)	0.73 (0.06, 12.62)	0.55 (0.09, 4.65)	0.69 (0.09, 14.59)		
2.49 (0.29, 24.91)	1.12 (0.23, 8.07)	0.58 (0.12, 6.07)	2.68 (0.24, 74.04)	1.08 (0.20, 11.04)	1.16 (0.12, 15.57)	0.78 (0.22, 4.00)	1.03 (0.14, 18.98)	1.49 (0.13, 15.91)	

*ABLC* Amphotericin B lipid complex, *AmB* Conventional amphotericin B, *L-AmB* Liposomal amphotericin B, *NAT* No antifungal treatment

^a^1 mg/kg/day; ^b^3 mg/kg/day

#### Fungal infection-related mortality

**Fig. 3:**
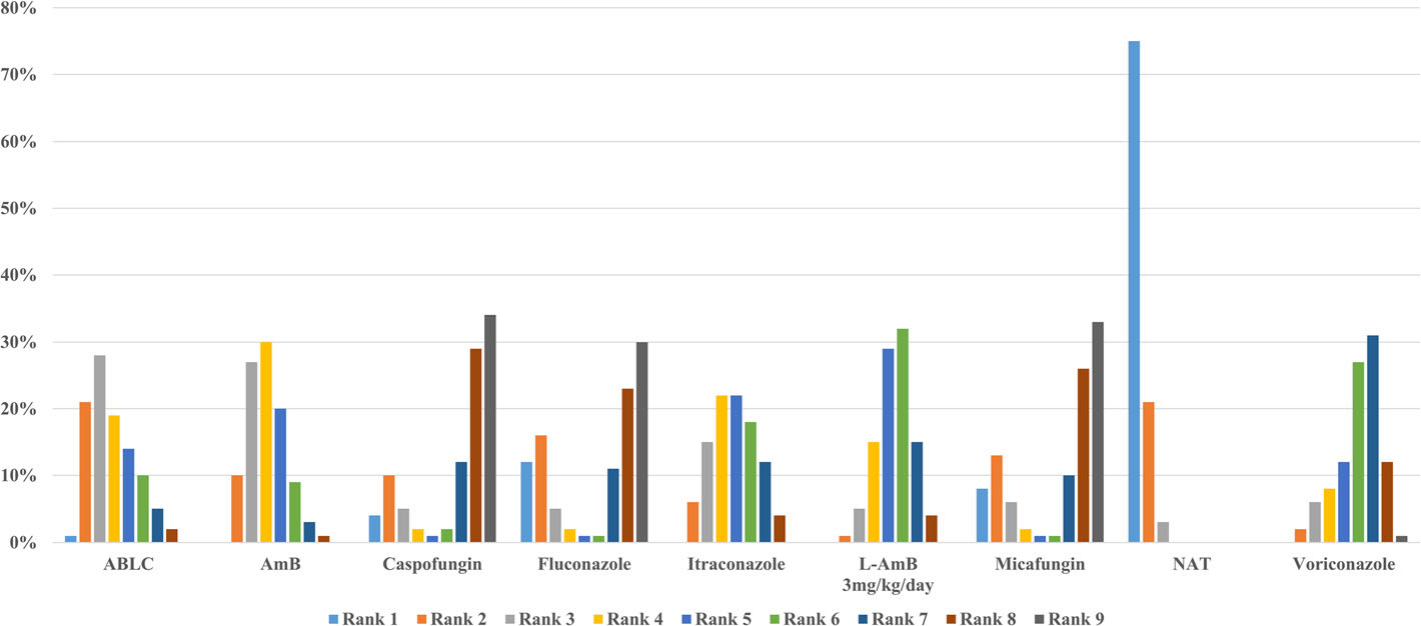
Rank probability plot of antifungals for fungal infection-related mortality. ABLC: Amphotericin B lipid complex; AmB: Conventional amphotericin B; L-AmB: Liposomal amphotericin B; NAT: No antifungal treatment. Rank 1 is worst, rank 9 is best

Ten studies (nine arms, 2747 patients) were included for fungal infection-related mortality (Additional file 2) [17, 19, 21–23, 26–29, 32]. According to the results of NMA, ABLC, AmB, itraconazole, L-AmB, and voriconazole had a significantly lower rate of fungal infection-related mortality than NAT, respectively (Additional file 1). From the rank probability plot, we found that caspofungin appeared to have the lowest rate of fungal infection-related mortality (Fig. 3).

#### Treatment response

**Fig. 4:**
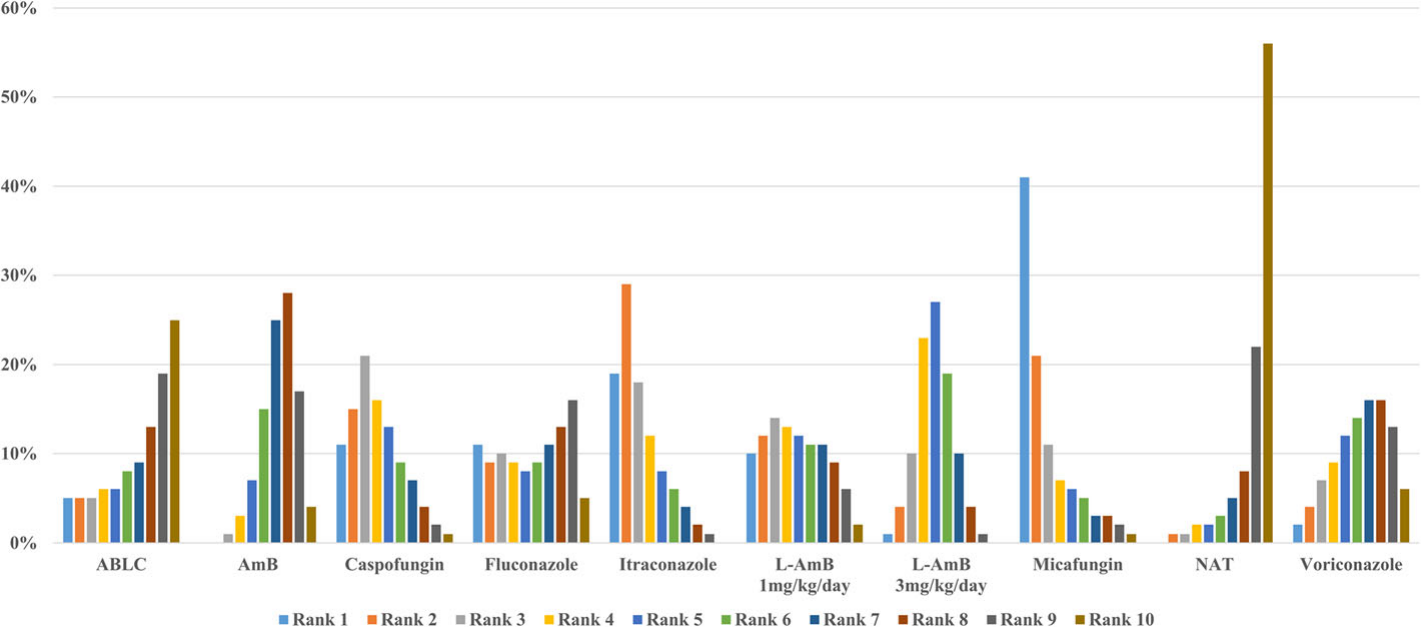
Rank probability plot of antifungals for treatment response. ABLC: Amphotericin B lipid complex; AmB: Conventional amphotericin B; L-AmB: Liposomal amphotericin B; NAT: No antifungal treatment Rank 1 is best, rank 10 is worst

Seventeen studies (ten arms, 4583 patients) were included for treatment response (Additional file 2) [17–32]. According to the results of NMA, there were no statistical differences among all agents (Additional file 1). From the rank probability plot, we could see that micafungin appeared to have the highest rate of treatment response (Fig. 4).

### Subgroup and sensitivity analysis

Subgroup analysis for proportion of allo-HSCT indicated that for patients with proportion of allo-HSCT less than 10%, there were no statistical difference among all agents regarding treatment response [17, 18, 21, 23, 25, 26, 29, 30, 33]. From the rank probability plot, fluconazole appeared to have the highest rate of treatment response (Additional file 2). No network of evidence for patients with proportion of allo-HSCT more than 10% and for other outcomes was constructed.

Subgroup analysis for ages indicated that for adults, itraconazole and micafungin had a significantly higher rate of treatment response than NAT. For children, there was no statistical difference among all agents for treatment response. From the rank probability plot, caspofungin appeared to have the highest treatment response rate for both adults and children (Additional file 2). No network of evidence for all-cause mortality and fungal infection-related mortality was constituted. Due to lack of data, azoles, micafungin and ABLC were not included in the subgroup analysis of children.

After excluding studies with any items under high risk of bias according to our quality appraisal [17–19, 22, 27, 31], we found that there was no statistical difference among all agents for all-cause mortality, fungal infection-related mortality and treatment response, respectively. Rank probability plot presented that caspofungin appeared to be the most effective agent for all-cause mortality, whereas micafungin tended to be superior for fungal infection-related mortality and treatment response (Additional file 2). Fluconazole was not included in the network due to lack of evidence.

### Inconsistency between direct and indirect effect

Node-splitting analysis revealed that there were not any statistical differences among direct, indirect, and combined effects were available for comparisons of all outcomes (Additional file 1).

## Discussion

Based upon our analysis of mortality and treatment response, echinocandins appear to be the most effective agents for empiric treatment of IFD in FN patients. A similar systematic review and NMA was published in 2011 [37], however it only included RCTs of adult patients regarding anti-mold agents and it included RCTs using irregular dosage and failed to include some RCTs. There has since been an additional three studies published comparing echinocandins to other therapies, allowing to us show mortality benefits of this class of antifungals in IFD. The results of this analysis highlight the necessity of empiric antifungal treatment for FN patients failing to respond to initial broad-spectrum antibacterial treatment and provide perspective on the selection of drug therapies.

Diagnosis of IFDs requires specific clinical manifestations (e.g. halo or crescent sign from chest computed tomography), mycological evidence (e.g. presence of fungal elements indicating a mold in sputum, plasma galactomannan antigen detected) or positive fungal culture from sterile sites. However, none of the diagnosis criteria for IFDs presents adequate sensitivity [8]. Therefore, in cases where neutropenic patients remain febrile despite days of broad-spectrum anti-bacterial treatment and absence of infection evidence, antifungal treatment should be given prior to diagnosis of IFDs. A 2008 systematic review indicated that empiric treatment did not decrease mortality significantly, but decreased IFDs (RR 0.25, 95% CI 0.12–0.54) [10]. Thus, empiric antifungal treatment has been recommended by the latest practice guidelines [12, 13]. Our NMA strongly supports the advantage of empiric antifungal treatment by indicating that five antifungal agents have a significant lower rate of fungal infection-related mortality than NAT, respectively, in spite of the poor precision of the results. For our primary outcome, reduction in all-cause mortality is difficult to achieve in RCTs with neutropenic patients, and the results of our study support this finding.

The echinocandins are broad-spectrum, parenteral antifungals recommended as initial therapy in hospitalized patients with candidemia [11]. Echinocandins are much more likely to be effective against *C. glabrata* and *C. krusei* than triazoles due to fungicidal activity and universally higher probability of organism susceptibility [38]. Our results indicate that caspofungin or micafungin rank highest for reducing mortality and improving treatment response outcomes. The results were stable after excluding studies with high risk of bias. Caspofungin and micafungin are similar in chemical structure, antifungal spectrum and pharmacokinetic profile [39]. Although direct comparison between echinocandins as empiric antifungal treatment is lacking, an RCT comparing caspofungin with micafungin in the treatment of candidiasis and aspergillosis revealed that the efficacy of caspofungin and micafungin was similar [40]. However, of 17 RCTs included in our NMA, only 2 RCTs studied micafungin with a low sample size of 242 patients [23]. Therefore, conclusions about micafungin should be drawn with caution. In conclusion, echinocandins, especially caspofungin, have an advantage of empiric antifungal treatment compared with other antifungals based upon efficacy outcomes.

Selection of agents for the treatment of serious fungal infections is primarily based upon efficacy, safety, costs, available formulations, and the potential for drug interactions. Echinocandins have a favorable profile of safety and drug interactions compared to triazoles. Echinocandins are fungistatic against Aspergillus, where some triazoles or amphotericin formulations would usually be favored. Echinocandins also have limited activity against mucorales and *Fusarium* species compared to other agents. Depending on the setting, echinocandins are generally more cost-effective [41–45]. Despite amphotericin having the broadest spectrum of activity, intolerance has been a major reason for the development and use of echinocandins and triazoles. L-AmB and ABLC significantly reduce the probability of nephrotoxicity when compared to AmB. However, these agents still have a notably greater risk of adverse effects, including infusion-related reactions, neutropenia, and electrolyte abnormalities compared to the other antifungal agents. Drug interactions are a major issue for triazoles, and in some instances the suboptimal bioavailability (posaconazole and itraconazole) can also be important. The need for blood level monitoring could be considered an advantage or disadvantage. The azoles are the only agents available IV and orally, thus ease of administration. Our meta-analysis supports the IDSA and ECIL Guidelines’ recommendations which indicate a high level of evidence for echinocandins for empiric antifungal therapy [11–13].

The necessity of stratifying FN patients according to their risk of fungal infection remains controversial. In our study, many of the RCTs conducted a stratified randomization based on fungal infection risk [17, 18, 21, 23, 26, 29, 30]. The definition of high and low risk was inconsistent among studies. Our results of NMA indicated that for treatment response, fluconazole was the most effective agent in patients not undergoing allo-HSCT. The spectrum of fluconazole is not as broad as other antifungals mentioned above. Although not recommended for empiric antifungal therapy in FN patients, fluconazole is regarded as an acceptable alternative for critically-ill nonneutropenic patients [11]. Supporting this, it appears that the most effective empiric antifungal therapy in FN patients may differ between high and low risk of fungal infection. In addition, it remains unclear the difference of empiric treatment between adult and pediatric patients according to our subgroup analysis.

There are several limitations in our NMA. First, NMA is an indirect comparison that is not able to substitute large, well-designed RCTs. Whereas due to lack of head-to-head studies, NMA is the optimal evaluation with available data. Second, the RCTs included in the current study failed to meet power secondary to being unable to include specified sample size. For instance, the statistical power was inadequate due to limited sample size for micafungin. Subsequently, although micafungin ranked high in many outcomes during NMA, conclusions were drawn with considerable caution. Third, quite a few RCTs were under high risk of bias due to lack of blinding outcome assessment or intention-to-treat, thus we conducted sensitivity analysis to overcome the weakness. Last, the studies spanned over a 27 year period and interventions since the 1980’s have continued to evolve and no single study would be considered to include a large number of subjects.

## Conclusions

In summary, our study provides a valuable reference for antifungal use as empiric treatment. More head-to-head studies are required in order to further facilitate decision making regarding the best empiric treatment alternative. Additionally, decision making should focus on the safety and cost effectiveness of such antifungals in parallel. Based on our meta- analysis, the echinocandins appear to be the most effective agents. However, individual patient and health system factors may influence the appropriate selection of empiric antifungals in neutropenic patients.

## Additional files

Additional file 1:
**Table S1.** Sample search strategy for MEDLINE.** Table S2.** The reason for excluding studies.** Table S3.** Raw data. **Table S4.** The network meta-analysis results (presented as odds ratio) for fungal infection-related mortality.** Table S5.** The network meta-analysis results (presented as odds ratio) for treatment response.** Table S6.** Node-splitting analysis for all-cause mortality.**Table S7.** Node-splitting analysis for treatment response. (DOCX 34 kb)
Additional file 2: Figure S1-20. (PDF 340 kb)

## Abbreviations

ABLC: amphotericin B lipid complex
allo-HSCT: Allogenic hematologic stem cell transplantation
AmB: Conventional amphotericin B
CI: Confidence intervals
ECIL: European conference on infections in leukemia
FDA: Food and drug administration
FN: Febrile neutropenic
HSCT: Hematopoietic stem cell transplantation
IDSA: Infectious diseases society of america
IFDs: Invasive fungal diseases
ITT: Intention-to-treatment
L-AmB: Liposomal amphotericin B
NAT: No antifungal treatment
NMA: Network meta-analysis
RCTs: Randomized controlled trials
RR: Risk ratio
