# Supplementary material for: Empiric treatment against invasive fungal diseases in febrile neutropenic patients: a systematic review and network meta-analysis
Source: BMC Infect Dis. 2017 Feb 20;17:159. doi: 10.1186/s12879-017-2263-6 (PMC5319086; doi:10.1186/s12879-017-2263-6)
Supplement: Additional file 1: — Table S1. Sample search strategy for MEDLINE. Table S2. The reason for excluding studies. Table S3. Raw data. Table S4. The network meta-analysis results (presented as odds ratio) for fungal infection-related mortality. Table S5. The network meta-analysis results (presented as odds ratio) for treatment response. Table S6. Node-splitting analysis for all-cause mortality.Table S7. Node-splitting analysis for treatment response. (DOCX 34 kb) [file 12879_2017_2263_MOESM1_ESM.docx]

**Additional file 1**

**Table S1-Sample search strategy for MEDLINE**

| 1 | "antifungal agents"[Pharmacological Action] OR "antifungal agents"[MeSH Terms] OR ("antifungal"[All Fields] AND "agents"[All Fields]) OR "antifungal agents"[All Fields] OR "antifungal"[All Fields] |
| --- | --- |
| 2 | "amphotericin b"[MeSH Terms] OR "amphotericin b"[All Fields] OR "amphotericin"[All Fields] |
| 3 | "azoles"[MeSH Terms] OR "azoles"[All Fields] |
| 4 | "triazoles"[MeSH Terms] OR "triazoles"[All Fields] |
| 5 | "fluconazole"[MeSH Terms] OR "fluconazole"[All Fields] |
| 6 | "itraconazole"[MeSH Terms] OR "itraconazole"[All Fields] |
| 7 | "voriconazole"[MeSH Terms] OR "voriconazole"[All Fields] |
| 8 | "posaconazole"[Supplementary Concept] OR "posaconazole"[All Fields] |
| 9 | "echinocandins"[MeSH Terms] OR "echinocandins"[All Fields] OR "echinocandin"[All Fields] |
| 10 | "caspofungin"[Supplementary Concept] OR "caspofungin"[All Fields] |
| 11 | "micafungin"[Supplementary Concept] OR "micafungin"[All Fields] |
| 12 | "anidulafungin"[Supplementary Concept] OR "anidulafungin"[All Fields] |
| 13 | 1 OR 2 OR 3 OR 4 OR 5 OR 6 OR 7 OR 8 OR 9 OR 10 OR 11 OR 12 |
| 14 | "fever"[MeSH Terms] OR "fever"[All Fields] OR "febrile"[All Fields] OR "pyrexia"[All Fields] |
| 15 | "neutropaenia"[All Fields] OR "neutropenia"[MeSH Terms] OR "neutropenia"[All Fields] OR neutropenic[All Fields] OR "leukopenia"[MeSH Terms] OR "leukopenia"[All Fields] OR "agranulocytosis"[MeSH Terms] OR "agranulocytosis"[All Fields] OR "granulocytopenia"[All Fields] OR granulocytopenic[All Fields] |
| 16 | 13 AND 14 AND 15 |
| 17 | “randomized controlled trial”[pt] |
| 18 | “controlled clinical trial”[pt] |
| 19 | “randomized”[tiab] |
| 20 | “placebo”[tiab] |
| 21 | “clinical trials as topic”[mesh: noexp] |
| 22 | “randomly”[tiab] |
| 23 | “trial”[ti] |
| 24 | 17 OR 18 OR 19 OR 20 OR 21 OR 22 OR 23 |
| 25 | “animals”[mh] NOT “humans”[mh] |
| 26 | 24 NOT 25 |
| 27 | 16 AND 26 |

**Table S2-The reason for excluding studies**

| **No.** | **Excluded studies** | **Reason for exclusion** |
| --- | --- | --- |
| 1 | Empiric therapy with fluconazole in granulocytopenic patients with carcinoma or leukemia. Japanese journal of antibiotics. 1994. 47(8): 1065-70. | Not in English or Chinese |
| 2 | Liposomal amphotericin B versus conventional amphotericin B in the empirical treatment of persistently febrile neutropenic patients. Journal of antimicrobial chemotherapy. 2002. 49(SUPL. S1): 81-6. | Duplicate publication |
| 3 | Micafungin versus voriconazole for empirical antifungal therapy in febrile neutropenic patients with acute myeloid leukemia: A randomized, controlled trial. Blood. 2010. 116(21). | Duplicate publication |
| 4 | Impact of alternate definitions of fever resolution on the composite endpoint in clinical trials of empirical antifungal therapy for neutropenic patients with persistent fever: analysis of results from the Caspofungin Empirical Therapy Study. Transplant infectious disease. 2006. 8(1): 31-7. | Duplicate publication |
| 5 | Review of comparative studies between conventional and liposomal amphotericin B (Ambisome) in neutropenic patients with fever of unknown origin and patients with systemic mycosis. Mycoses. 2000. 43(9-10): 325-32. | Duplicate publication |
| 6 | Empirical anti-fungal therapy in patients with hematological disease. Chinese Journal of Infection and Chemotherapy. 2007. 7(3): 187. | Not RCT |
| 7 | Caspofungin: in pediatric patients with fungal infections. Paediatr Drugs. 2009. 11(4): 259-69. | Not RCT |
| 8 | Lipid formulations of amphotericin B for empirical treatment of fever and neutropenia. Clin Infect Dis. 2002. 35(7): 896-7. | Not RCT |
| 9 | Intravenous itraconazole vs. amphotericin B deoxycholate for empirical antifungal therapy in patients with persistent neutropenic fever. Korean J Intern Med. 2006. 21(3): 165-72. | Not RCT |
| 10 | The efficacy of itraconazole against systemic fungal infections in neutropenic patients: A randomized comparative study with amphotericin B. MED KLIN. 1991. 86(SUPPL. 1): 11-3. | Wrong target population |
| 11 | Population pharmacokinetics of liposomal amphotericin B and caspofungin in allogeneic hematopoietic stem cell recipients. Antimicrob Agents Chemother. 2012. 56(1): 536-43. | No desired outcomes |
| 12 | Intravenous followed by oral itraconazole versus intravenous amphotericin B as empirical antifungal therapy for febrile neutropenic haematological cancer patients. Blood. 2002. 100(11/2): 42b. | Full texts unavailable |
| 13 | Amphotericin B and itraconazole as empirical antifungal therapy in children with acute leukemia with neutropenic fever [Abstract No. 4577]. Blood. 2005. 106(11 Part 1): 223b. | Full texts unavailable |
| 14 | Improved safety and efficacy of amBisome (liposomal amphotericin B) compared with amphotericin B in the empirical treatment of febrile neutropenic patients undergoing bone marrow transplantation. Blood. 1997. 90(10 Suppl 1 (Pt 1)): 420a, Abstract 1866. | Full texts unavailable |
| 15 | Intralipid does not decrease the toxicity of amphotericin B. Results of a randomized phase II trial in neutropenic patients with fever of unknown origin or pneumonia. Blood. 1996. 88(10 Suppl (Pt 1)): 502a, Abstract 1995. | Full texts unavailable |
| 16 | Early AmBisome in febrile neutropenia in patients with haematological disorders. Bone Marrow Transplant. 1994. 14 Suppl 5: S15-7. | Full texts unavailable |
| 17 | Administration of lipid-emulsion versus conventional amphotericin B in patients with neutropenia. Annals of pharmacotherapy. 1995. 29(12): 1197-201. | Full texts unavailable |
| 18 | Phase III randomised study of caspofungin acetate versus amphotereicin B liposomal in patients with persistent fever and neutropenia following treatment for cancer. Physician Data Query (PDQ). 2001. | Full texts unavailable |
| 19 | Comparison of micafungin and liposomal amphotericin b for empirical antifungal therapy in febrile neutropenic patients with hematological malignancies: A randomized controlled trial. Haematologica. 2015. 100: 135. | Full texts unavailable |
| 20 | Low-dose amphotericin B lipid complex vs. conventional amphotericin B for empirical antifungal therapy of neutropenic fever in patients with hematologic malignancies--a randomized, controlled trial. Eur J Haematol. 2004. 72(5): 342-7. | Dosage not referred to package inserts |
| 21 | A randomized comparison of fluconazole with amphotericin B as empiric anti-fungal agents in cancer patients with prolonged fever and neutropenia. American journal of medicine. 1998. 105(6): 478-83. | Dosage not referred to package inserts |
| 22 | A multicenter, randomized trial of fluconazole versus amphotericin B for empiric antifungal therapy of febrile neutropenic patients with cancer. American journal of medicine. 2000. 108(4): 282-9. | Dosage not referred to package inserts |
| 23 | Randomized, double-blind clinical trial of amphotericin B colloidal dispersion vs. amphotericin B in the empirical treatment of fever and neutropenia. Clinical infectious diseases. 1998. 27(2): 296-302. | Formulation of amphotericin B not approved by U S Food and Drug Administration |
| 24 | Use of amphotericin B colloidal dispersion in children. J Pediatr Hematol Oncol. 2000. 22(3): 242-6. | Formulation of amphotericin B not approved by U S Food and Drug Administration |

**Table S3-Raw data**

| **References** | **All-cause mortality** | | | **Fungal infection-related mortality** | | | **Treatment response** | | |
| --- | --- | --- | --- | --- | --- | --- | --- | --- | --- |
|  | **A** | **B** | **C** | **A** | **B** | **C** | **A** | **B** | **C** |
| Boogaerts 2001[17] | 19/179^a^ | 25/181^b^ | / | 2/179^a^ | 4/181^b^ | / | 84/179^a^ | 68/181^b^ | / |
| Caselli 2012[18] | NR | NR | / | NR | NR | / | 22/25^c^ | 26/31^d^ | / |
|  | NR | NR | / | NR | NR | / | 12/15^c^ | 16/17^d^ | 14/16^e^ |
| Groll 2010[19] | 0/18^d^ | 1/20^c^ | / | 0/18^d^ | 0/20^c^ | / | 14/18^d^ | 15/20^c^ | / |
| Jadhav 2012[20] | 3/23^c^ | 2/20^b^ | 3/22^f^ | NR | NR | NR | 13/23^c^ | 12/20^b^ | 15/22^f^ |
| Jeong 2016[33] | 6/73^g^ | 8/75^a^ | / | NR | NR | / | 47/73^g^ | 43/75^a^ | / |
| Maertens 2010[21] | 0/15^d^ | 0/7^c^ | / | 0/15^d^ | 0/7^c^ | / | 9/15^d^ | 0/7^c^ | / |
|  | 0/41^d^ | 0/18^c^ | / | 0/41^d^ | 0/18^c^ | / | 17/41^d^ | 8/18^c^ | / |
| Meunier 1989[22] | 11/68^b^ | 14/64^e^ | / | 0/68^b^ | 4/64^e^ | / | 38/57^b^ | 24/51^e^ | / |
|  |  |  |  |  |  |  | 9/11^b^ | 10/13^e^ | / |
| Oyake 2015[23] | 1/49^g^ | 0/45^h^ | / | 0/49^g^ | 0/45^h^ | / | 32/49^g^ | 28/45^h^ | / |
| Prentice 1997[24] | NR | NR | / | NR | NR | / | 18/39^b^ | 30/47^c^ | 23/47^f^ |
|  | NR | NR | / | NR | NR | / | 31/61^b^ | 45/71^c^ | 45/70^f^ |
| Schiel 2006[25] | 0/54^e^ | 1/56^i^ | / | NR | NR | / | 30/54^e^ | 35/56^i^ | / |
| Schuler 2007[26] | 14/81^a^ | 13/81^b^ | / | 1/81^a^ | 1/81^b^ | / | 16/26^a^ | 8/24^b^ | / |
|  |  |  |  |  |  |  | 34/55^a^ | 26/57^b^ | / |
| Viscoli 1996[27] | 3/56^i^ | 2/56^b^ | / | 0/56^i^ | 0/56^b^ | / | 42/56^i^ | 37/56^b^ | / |
| Walsh 1999[28] | 25/343^c^ | 36/344^b^ | / | 4/343^c^ | 11/344^b^ | / | 172/343^c^ | 170/344^b^ | / |
| Walsh 2002[29] | 33/415^h^ | 25/422^c^ | / | 1/415^h^ | 2/422^c^ | / | 46/143^h^ | 42/141^c^ | / |
|  |  |  |  |  |  |  | 62/272^h^ | 87/281^c^ | / |
| Walsh 2004[30] | 41/556^d^ | 58/539^c^ | / | NR | NR | / | 63/146^d^ | 46/122^c^ | / |
|  |  |  |  |  |  |  | 127/410^d^ | 135/417^c^ | / |
| Wang 2007[31] | 2/32^d^ | 0/28^c^ | / | NR | NR | / | 21/32^d^ | 19/28^c^ | / |
| Wingard 2000[32] | 5/85^c^ | 11/78^j^ | / | 1/85^c^ | 3/78^j^ | / | 34/85^c^ | 26/78^j^ | / |

^a^Itraconazole; ^b^Conventional amphotericin B; ^c^Liposomal amphotericin B 3 mg/kg/day; ^d^Caspofungin; ^e^No antifungal treatment; ^f^Liposomal amphotericin B 1 mg/kg/day; ^g^Micafungin; ^h^Voriconazole; ^i^Fluconazole; ^j^Amphotericin B lipid complex

**Table S4-The network meta-analysis results (presented as odds ratio) for fungal infection-related mortality**

| **ABLC** |  |  |  |  |  |  |  |  |
| --- | --- | --- | --- | --- | --- | --- | --- | --- |
| 1.49 (0.02, 219.67) | **AmB** |  |  |  |  |  |  |  |
| 1.85*10^7^ (0.00, 2.94*10^29^) | 1.12*10^7^ (0.00, 1.78*10^29^) | **Caspofu-ngin** |  |  |  |  |  |  |
| 1.58*10^5^ (0.00, 6.17*10^29^) | 9.57*10^4^ (0.00, 2.81*10^29^) | 0.01 (0.00, 5.77*10^28^) | **Fluconaz-ole** |  |  |  |  |  |
| 2.71 (0.01, 629.37) | 1.70 (0.14, 20.34) | 0.00 (0.00, 1.30*10^10^) | 0.00 (0.00, 3.52*10^16^) | **Itracona-zole** |  |  |  |  |
| 4.41 (0.15, 293.77) | 2.95 (0.18, 53.53) | 0.00 (0.00, 1.96*10^10^) | 0.00 (0.00, 7.57*10^16^) | 1.78 (0.04, 80.04) | **L-AmB^a^** |  |  |  |
| 5.57*10^6^ (0.00, 3.69*10^32^) | 3.61*10^6^ (0.00, 1.40*10^32^) | 0.36 (0.00, 3.64*10^30^) | 80.54 (0.00, 2.08*10^35^) | 2.09*10^6^ (0.00, 9.84*10^31^) | 1.23*10^6^ (0.00, 4.94*10^31^) | **Micafun-gin** |  |  |
| 0.00 (0.00, 0.58) | 0.00 (0.00, 0.11) | 0.00 (0.00, 3101.94) | 0.00 (0.00, 1.31*10^10^) | 0.00 (0.00, 0.09) | 0.00 (0.00, 0.05) | 0.00 (0.00, 4.29*10^6^) | **NAT** |  |
| 12.14 (0.08, 4279.94) | 8.13 (0.08, 1289.95) | 0.00 (0.00, 7.10*10^10^) | 0.00 (0.00, 1.99*10^17^) | 4.81 (0.03, 1318.47) | 2.55 (0.07, 187.12) | 0.00 (0.00, 5.27*10^13^) | 1.67*10^9^ (25.88, 4.12*10^26^) | **Voricona-zole** |

ABLC: Amphotericin B lipid complex; AmB: Conventional amphotericin B; L-AmB: Liposomal amphotericin B; NAT: No antifungal treatment

^a^3 mg/kg/day

**Table S5-The network meta-analysis results (presented as odds ratio) for treatment response**

| **ABLC** |  |  |  |  |  |  |  |  |  |
| --- | --- | --- | --- | --- | --- | --- | --- | --- | --- |
| 0.93 (0.35, 2.53) | **AmB** |  |  |  |  |  |  |  |  |
| 0.67 (0.25, 1.79) | 0.72 (0.38, 1.29) | **Caspof-ungin** |  |  |  |  |  |  |  |
| 0.80 (0.23, 2.65) | 0.85 (0.38, 1.83) | 1.20 (0.46, 3.05) | **Flucona-zole** |  |  |  |  |  |  |
| 0.58 (0.20, 1.71) | 0.63 (0.37, 1.04) | 0.88 (0.40, 1.93) | 0.73 (0.29, 1.94) | **Itracon-azole** |  |  |  |  |  |
| 0.72 (0.24, 2.07) | 0.78 (0.41, 1.40) | 1.08 (0.51, 2.23) | 0.89 (0.34, 2.41) | 1.24 (0.56, 2.68) | **L-AmB^a^** |  |  |  |  |
| 0.75 (0.31, 1.77) | 0.80 (0.52, 1.24) | 1.11 (0.75, 1.77) | 0.93 (0.40, 2.33) | 1.27 (0.68, 2.47) | 1.03 (0.58, 1.91) | **L-AmB^b^** |  |  |  |
| 0.53 (0.17, 1.83) | 0.58 (0.26, 1.29) | 0.81 (0.32, 2.16) | 0.67 (0.23, 2.13) | 0.92 (0.45, 1.94) | 0.75 (0.29, 1.96) | 0.72 (0.32, 1.65) | **Micafu-ngin** |  |  |
| 1.34 (0.40, 4.13) | 1.41 (0.68, 2.74) | 2.00 (0.81, 4.60) | 1.66 (0.78, 3.56) | 2.25 (0.95, 5.27) | 1.83 (0.72, 4.48) | 1.78 (0.78, 3.71) | 2.48 (0.82, 6.63) | **NAT** |  |
| 0.85 (0.29, 2.37) | 0.92 (0.43, 1.76) | 1.27 (0.59, 2.65) | 1.05 (0.38, 3.07) | 1.45 (0.66, 3.06) | 1.18 (0.50, 2.67) | 1.15 (0.59, 2.03) | 1.57 (0.68, 3.40) | 0.65 (0.25, 1.67) | **Voricon-azole** |

ABLC: Amphotericin B lipid complex; AmB: Conventional amphotericin B; L-AmB: Liposomal amphotericin B; NAT: No antifungal treatment

^a^1 mg/kg/day; ^b^3 mg/kg/day

**Table S6-Node-splitting analysis for all-cause mortality**

| **Name** | **Direct Effect** | **Indirect Effect** | **Overall** | **P-Value** |
| --- | --- | --- | --- | --- |
| AmB, fluconazole | 0.51 (-1.90, 3.01) | 9.77 (-0.84, 32.84) | 0.82 (-1.15, 3.29) | 0.14 |
| AmB, itraconazole | -0.13 (-1.27, 1.04) | 15.78 (-0.59, 50.66) | -0.06 (-1.01, 1.15) | 0.07 |
| AmB, L-AmB | -0.28 (-1.45, 1.13) | -15.01 (-48.30, -0.26) | -0.37 (-1.54, 0.84) | 0.05 |
| AmB, NAT | 0.34 (-1.33, 2.01) | -15.85 (-40.06, 0.23) | 0.24 (-1.53, 1.70) | 0.05 |
| Fluconazole, NAT | -13.12 (-41.29, 0.26) | -0.10 (-3.06, 2.79) | -0.66 (-3.55, 2.06) | 0.07 |
| Itraconazole, micafungin | -0.32 (-2.18, 1.57) | 9.60 (-0.99, 23.55) | -0.03 (-1.58, 1.78) | 0.08 |
| L-AmB, voriconazole | 0.31 (-1.32, 1.88) | -16.10 (-40.05, 0.23) | 0.24 (-1.39, 1.49) | 0.05 |
| Micafungin, voriconazole | -14.62 (-46.08, 0.03) | 0.53 (-2.36, 3.56) | -0.03 (-2.94, 1.96) | 0.05 |

AmB: Conventional amphotericin B; L-AmB: Liposomal amphotericin B 3 mg/kg/day; NAT: No antifungal treatment

**Table S7-Node-splitting analysis for treatment response**

| **Name** | **Direct Effect** | **Indirect Effect** | **Overall** | **P-Value** |
| --- | --- | --- | --- | --- |
| AmB, fluconazole | 0.44 (-0.54, 1.42) | -0.22 (-1.44, 1.02) | 0.16 (-0.61, 0.97) | 0.39 |
| AmB, itraconazole | 0.54 (0.02, 1.11) | -0.23 (-1.74, 1.34) | 0.46 (-0.04, 1.00) | 0.31 |
| AmB, L-AmB | 0.19 (-0.29, 0.68) | 0.56 (-0.80, 1.79) | 0.22 (-0.22, 0.65) | 0.58 |
| AmB, NAT | -0.69 (-1.58, 0.18) | 0.17 (-0.91, 1.29) | -0.34 (-1.01, 0.38) | 0.21 |
| Caspofungin, NAT | -0.10 (-1.91, 1.95) | -0.75 (-1.75, 0.11) | -0.69 (-1.53, 0.21) | 0.48 |
| Fluconazole, NAT | -0.32 (-1.21, 0.62) | -1.01 (-2.25, 0.24) | -0.51 (-1.27, 0.25) | 0.36 |
| Itraconazole, micafungin | 0.28 (-0.54, 1.20) | -0.42 (-1.94, 0.94) | 0.09 (-0.66, 0.79) | 0.30 |
| L-AmB, NAT | 0.12 (-1.68, 2.33) | -0.70 (-1.59, 0.21) | -0.58 (-1.31, 0.25) | 0.43 |
| L-AmB, voriconazole | -0.22 (-0.90, 0.51) | 0.56 (-1.01, 2.11) | -0.14 (-0.71, 0.53) | 0.33 |
| Micafungin, voriconazole | -0.09 (-1.10, 0.88) | -0.89 (-2.20, 0.36) | -0.45 (-1.22, 0.38) | 0.29 |

AmB: Conventional amphotericin B; L-AmB: Liposomal amphotericin B 3 mg/kg/day; NAT: No antifungal treatment
