# Supplementary material for: Empiric treatment against invasive fungal diseases in febrile neutropenic patients: a systematic review and network meta-analysis
Source: BMC Infect Dis. 2017 Feb 20;17:159. doi: 10.1186/s12879-017-2263-6 (PMC5319086; doi:10.1186/s12879-017-2263-6)
Supplement: Additional file 2: Figure S1-20. — (PDF 340 kb) [file 12879_2017_2263_MOESM2_ESM.pdf]

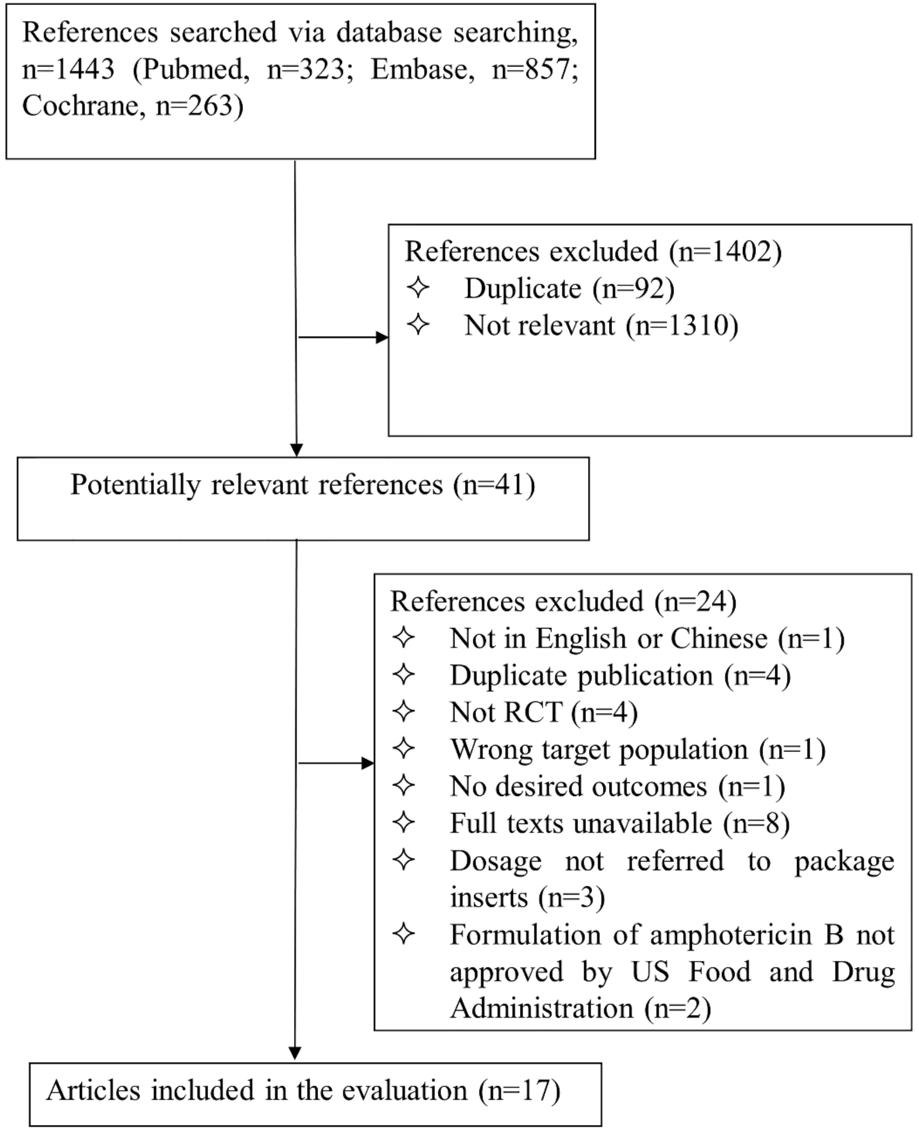

Figure S1. Flow chart of selection process

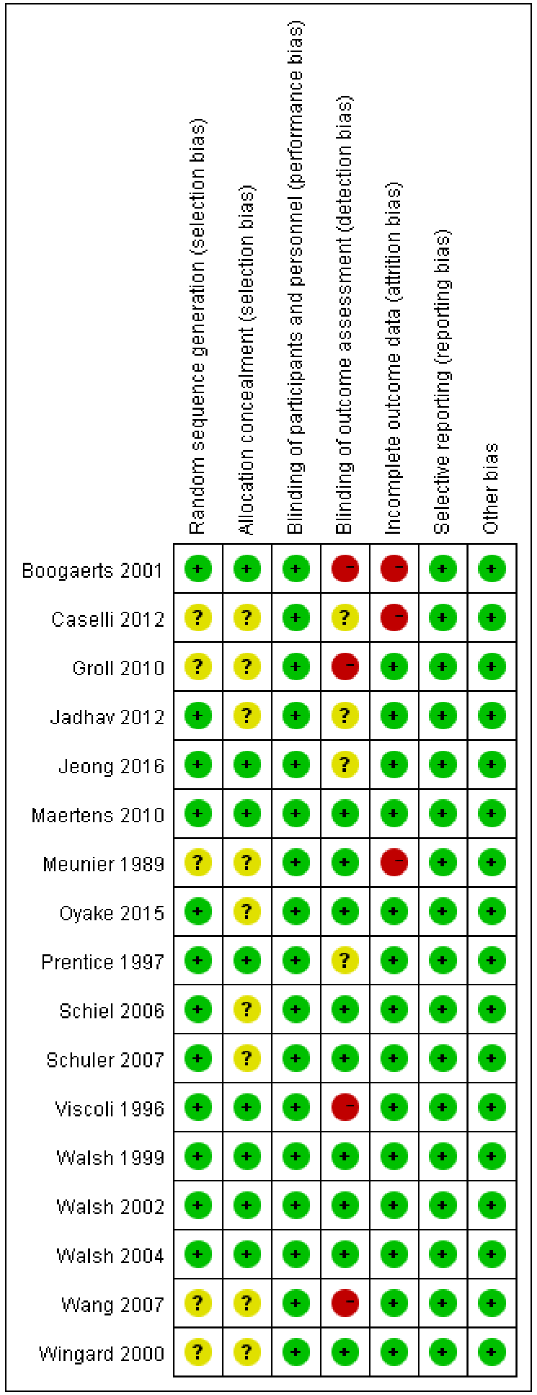

Figure S2. Risk of bias summary: review authors' judgements about each risk of bias item for each included study

Boogaerts 2001[17]; Caselli 2012[18]; Groll 2010[19]; Jadhav 2012[20]; Maertens 2010[21]; Meunier 1989[22]; Oyake 2015[23]; Prentice 1997[24]; Schiel 2006[25]; Schuler 2007[26]; Viscoli 1996[27]; Walsh 1999[28]; Walsh 2002[29]; Walsh 2004[30]; Wang 2007[31]; Wingard 2000[32]; Jeong 2016[33]

	Itraconazole		AmB			Risk Ratio
Study or Subgroup	Events	Total	Events	Total	Weight	M-H, Random, 95% CI
Boogaerts 2001	19	179	25	181	60.3%	0.77 [0.44, 1.34]
Schuler 2007	14	81	13	81	39.7%	1.08 [0.54, 2.15]
**Total (95% CI)**		**260**		**262**	**100.0%**	**0.88 [0.57, 1.36]**
Total events	33		38			
Heterogeneity: Tau² = 0.00; Chi² = 0.56, df = 1 (P = 0.46); I² = 0%						
Test for overall effect: Z = 0.58 (P = 0.56)						

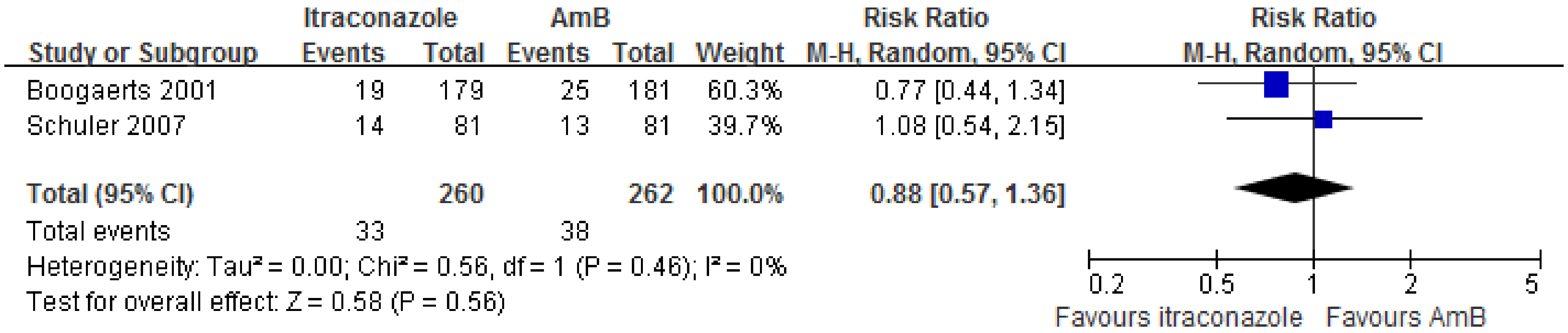

Figure S3. Forest plot of itraconazole versus conventional amphotericin B on all-cause mortality
AmB: Conventional amphotericin B

Study or Subgroup	Itraconazole Events	Itraconazole Total	AmB Events	AmB Total	Weight	Risk Ratio M-H, Random, 95% CI
Boogaerts 2001	2	179	4	181	72.8%	0.51 [0.09, 2.73]
Schuler 2007	1	81	1	81	27.2%	1.00 [0.06, 15.72]
**Total (95% CI)**		**260**		**262**	**100.0%**	**0.61 [0.14, 2.56]**
Total events	3		5			

Heterogeneity: Tau² = 0.00; Chi² = 0.17, df = 1 (P = 0.68); I² = 0%

Test for overall effect: Z = 0.68 (P = 0.50)

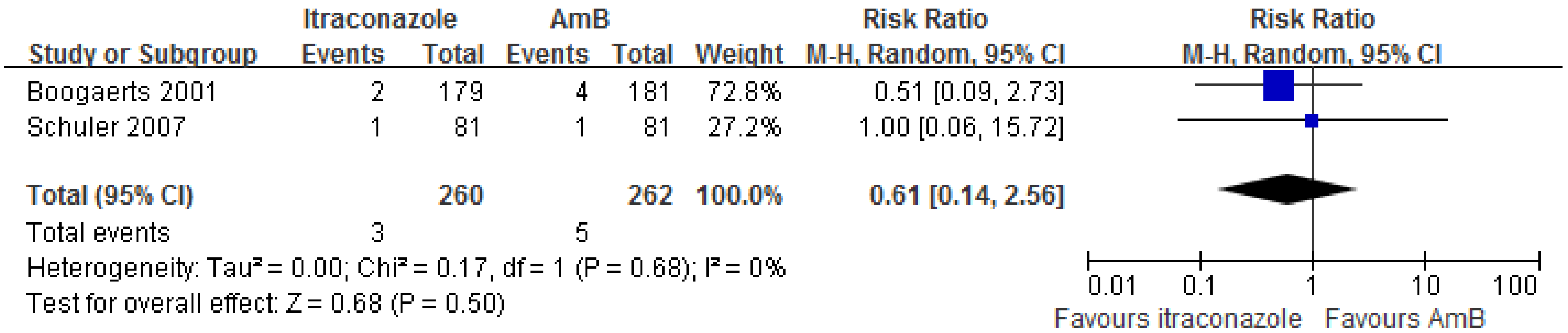

Figure S4. Forest plot of itraconazole versus conventional amphotericin B on fungal infection-related mortality

AmB: Conventional amphotericin B

Study or Subgroup	Itraconazole Events	Itraconazole Total	AmB Events	AmB Total	Weight	Risk Ratio M-H, Random, 95% CI
Boogaerts 2001	84	179	68	181	61.5%	1.25 [0.98, 1.59]
Schuler 2007	50	81	34	81	38.5%	1.47 [1.08, 2.00]
**Total (95% CI)**		**260**		**262**	**100.0%**	**1.33 [1.10, 1.61]**
Total events	134		102			

Heterogeneity: Tau² = 0.00; Chi² = 0.67, df = 1 (P = 0.41); I² = 0%

Test for overall effect: Z = 2.92 (P = 0.003)

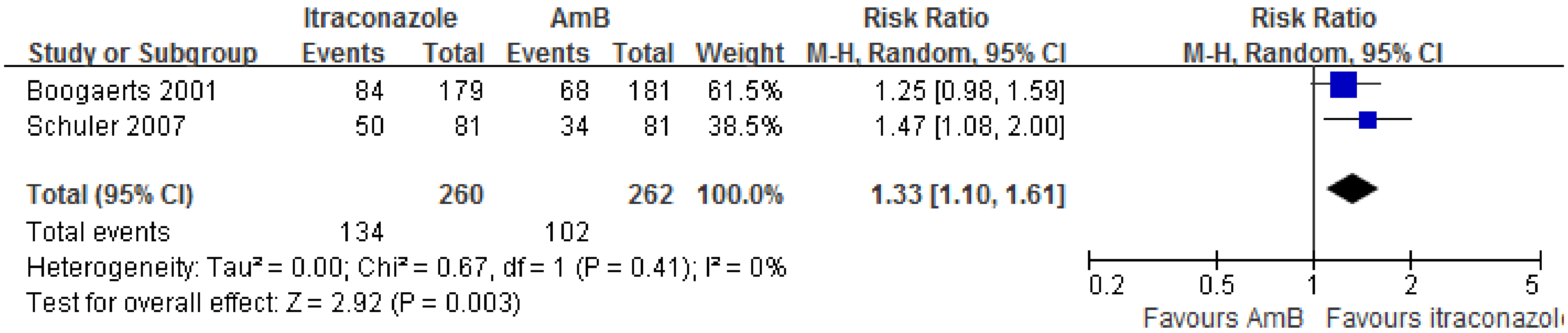

Figure S5. Forest plot of itraconazole versus conventional amphotericin B on treatment response

AmB: Conventional amphotericin B

	L-AmB 3 mg/kg/day		Caspofungin			Risk Ratio
**Study or Subgroup**	**Events**	**Total**	**Events**	**Total**	**Weight**	**M-H, Random, 95% CI**
Groll 2010	1	20	0	18	1.4%	2.71 [0.12, 62.70]
Maertens 2010	0	25	0	56		Not estimable
Walsh 2004	58	539	41	556	97.0%	1.46 [1.00, 2.14]
Wang 2007	0	28	2	32	1.6%	0.23 [0.01, 4.55]
**Total (95% CI)**		**612**		**662**	**100.0%**	**1.43 [0.98, 2.08]**
Total events	59		43			

Heterogeneity: Tau² = 0.00; Chi² = 1.62, df = 2 (P = 0.44); I² = 0%
Test for overall effect: Z = 1.86 (P = 0.06)

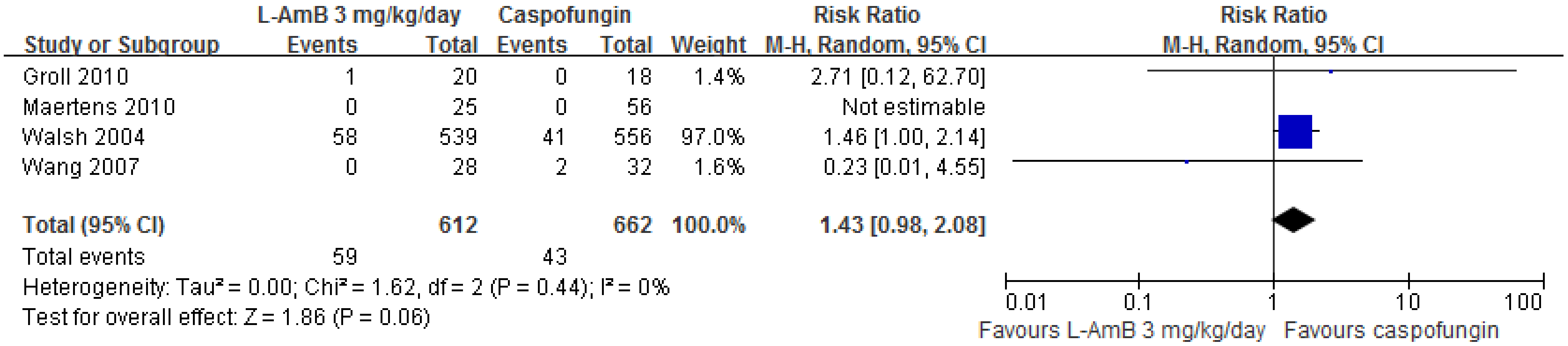

Figure S6. Forest plot of liposomal amphotericin B 3 mg/kg/day versus caspofungin on all-cause mortality
L-AmB: Liposomal amphotericin B

Study or Subgroup	L-AmB 3 mg/kg/day Events	L-AmB 3 mg/kg/day Total	Caspofungin Events	Caspofungin Total	Weight	Risk Ratio M-H, Random, 95% CI
Caselli 2012 High risk	22	25	26	31	24.8%	1.05 [0.85, 1.30]
Caselli 2012 Low risk	12	15	16	17	14.2%	0.85 [0.64, 1.12]
Groll 2010	15	20	14	18	8.9%	0.96 [0.68, 1.37]
Maertens 2010	8	25	26	56	2.7%	0.69 [0.36, 1.30]
Walsh 2004	181	539	190	556	40.6%	0.98 [0.83, 1.16]
Wang 2007	19	28	21	32	8.7%	1.03 [0.72, 1.48]
**Total (95% CI)**		**652**		**710**	**100.0%**	**0.97 [0.87, 1.08]**
Total events	257		293			

Heterogeneity: Tau² = 0.00; Chi² = 2.65, df = 5 (P = 0.75); I² = 0%
Test for overall effect: Z = 0.54 (P = 0.59)

Risk Ratio
M-H, Random, 95% CI

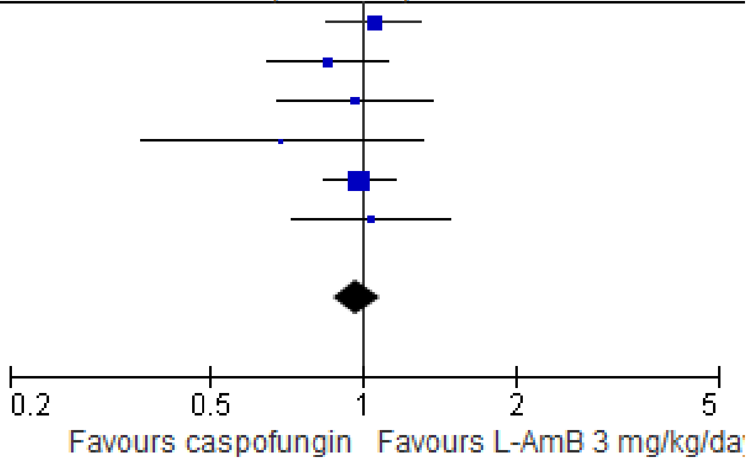

Figure S7. Forest plot of liposomal amphotericin B 3 mg/kg/day versus caspofungin on treatment response
L-AmB: Liposomal amphotericin B

Study or Subgroup	L-AmB 3 mg/kg/day Events	L-AmB 3 mg/kg/day Total	AmB Events	AmB Total	Weight	Risk Ratio M-H, Random, 95% CI
Jadhav 2012	3	23	2	20	7.7%	1.30 [0.24, 7.04]
Walsh 1999	25	343	36	344	92.3%	0.70 [0.43, 1.13]
**Total (95% CI)**		**366**		**364**	**100.0%**	**0.73 [0.46, 1.17]**
Total events	28		38			

Heterogeneity: Tau² = 0.00; Chi² = 0.49, df = 1 (P = 0.48); I² = 0%

Test for overall effect: Z = 1.31 (P = 0.19)

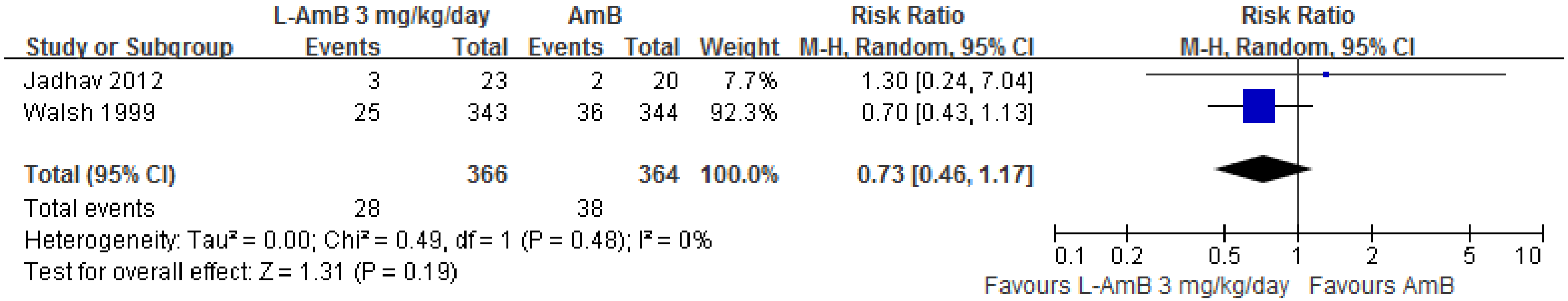

Figure S8. Forest plot of liposomal amphotericin B 3 mg/kg/day versus conventional amphotericin B on all-cause mortality

L-AmB: Liposomal amphotericin B; AmB: Conventional amphotericin B

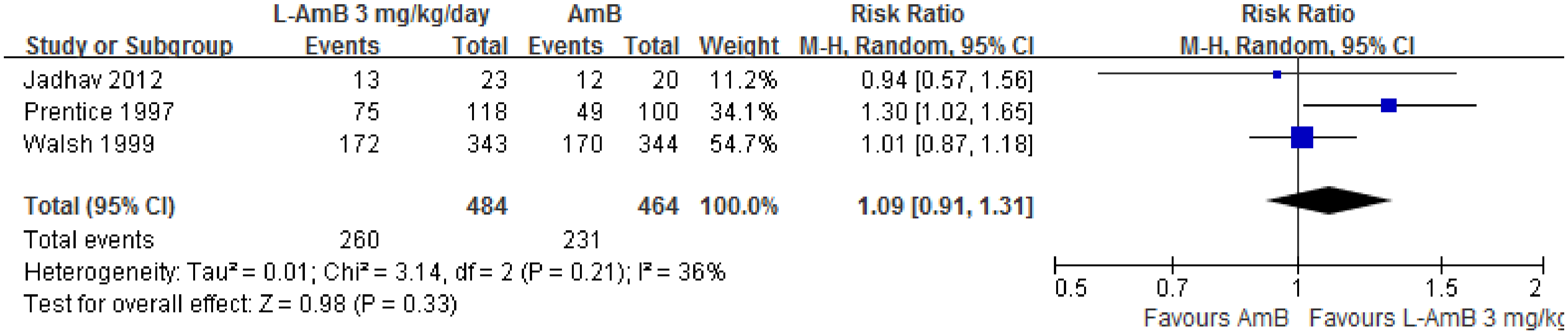

Figure S9. Forest plot of liposomal amphotericin B 3 mg/kg/day versus conventional amphotericin B on treatment response

L-AmB: Liposomal amphotericin B; AmB: Conventional amphotericin B

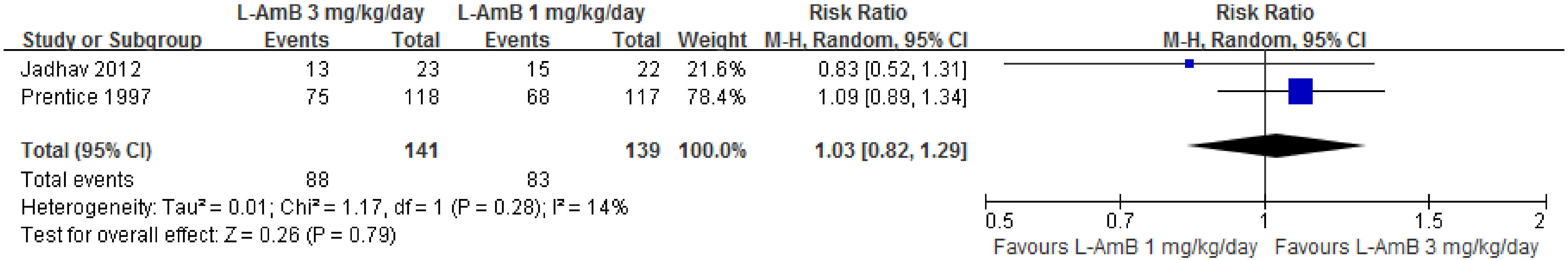

Figure S10. Forest plot of liposomal amphotericin B 3 mg/kg/day versus liposomal amphotericin B 1 mg/kg/day on treatment response

L-AmB: Liposomal amphotericin B

Study or Subgroup	AmB Events	AmB Total	L-AmB 1 mg/kg/day Events	L-AmB 1 mg/kg/day Total	Weight	Risk Ratio M-H, Random, 95% CI
Jadhav 2012	12	20	15	22	23.3%	0.88 [0.56, 1.39]
Prentice 1997	49	100	68	117	76.7%	0.84 [0.66, 1.09]
**Total (95% CI)**		**120**		**139**	**100.0%**	**0.85 [0.68, 1.06]**
Total events	61		83			

Heterogeneity: Tau² = 0.00; Chi² = 0.03, df = 1 (P = 0.87); I² = 0%
Test for overall effect: Z = 1.43 (P = 0.15)

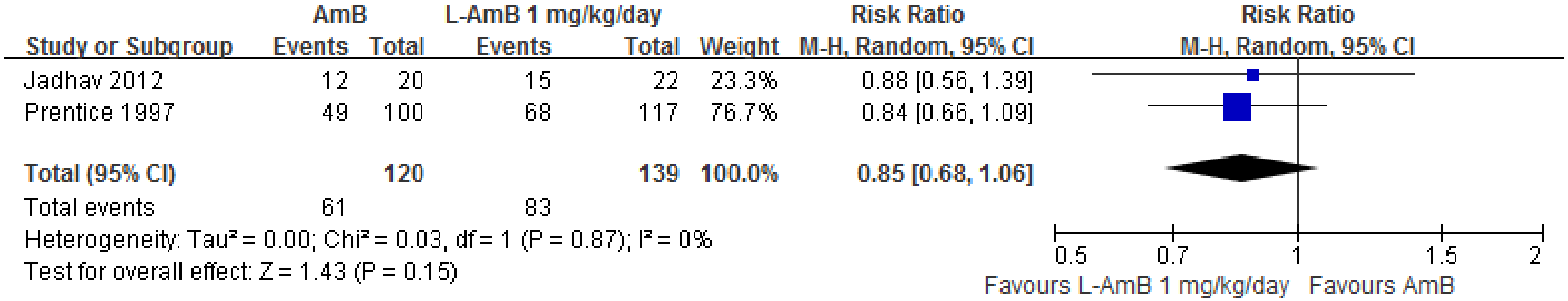

Figure S11. Forest plot of conventional amphotericin B versus liposomal amphotericin B 1 mg/kg/day versus on treatment response
AmB: Conventional amphotericin B; L-AmB: Liposomal amphotericin B

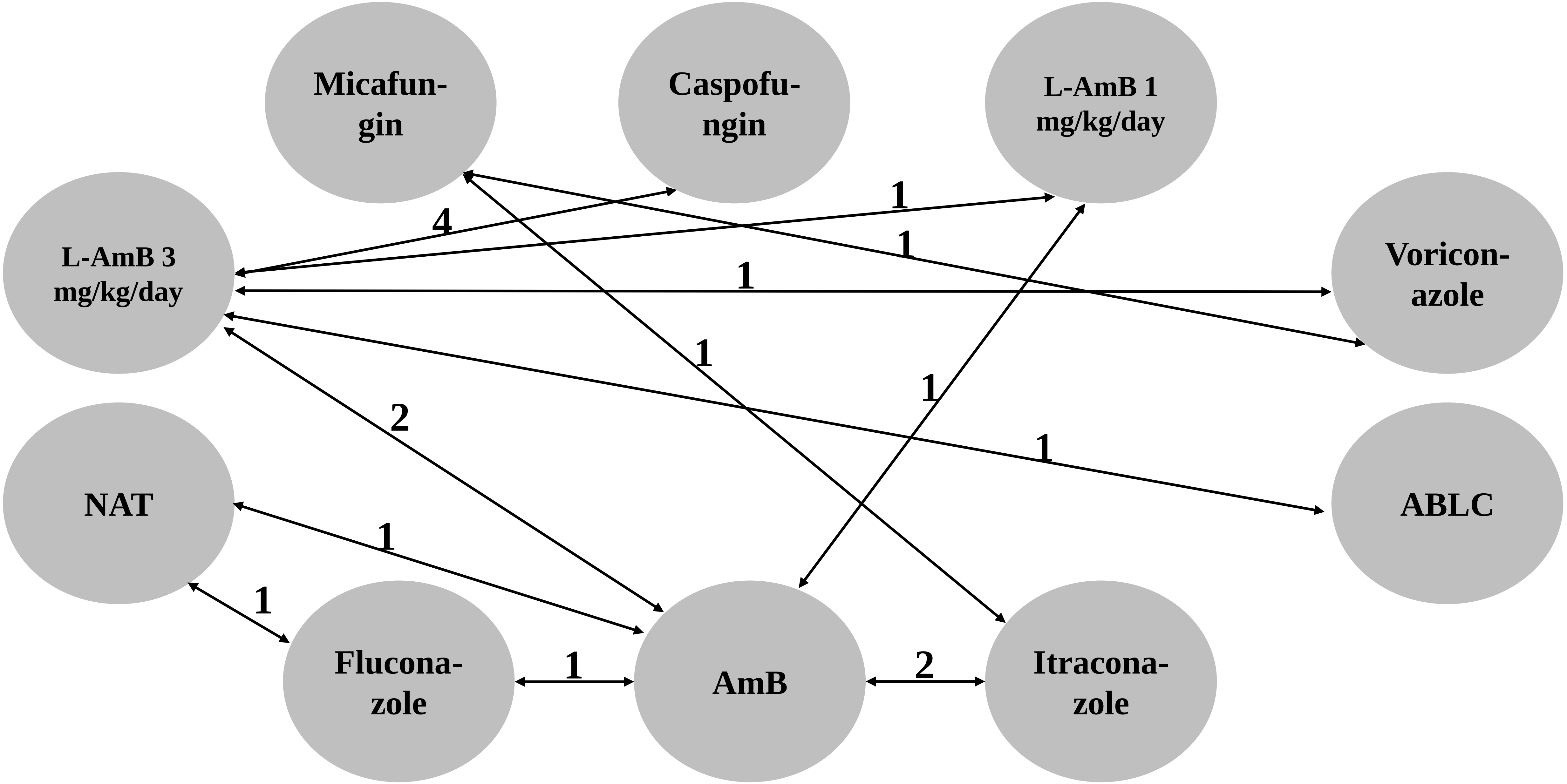

Figure S12. A schematic of the network of evidence for all-cause mortality

ABLC: Amphotericin B lipid complex; AmB: Conventional amphotericin B; L-AmB: Liposomal amphotericin B; NAT: No antifungal treatment

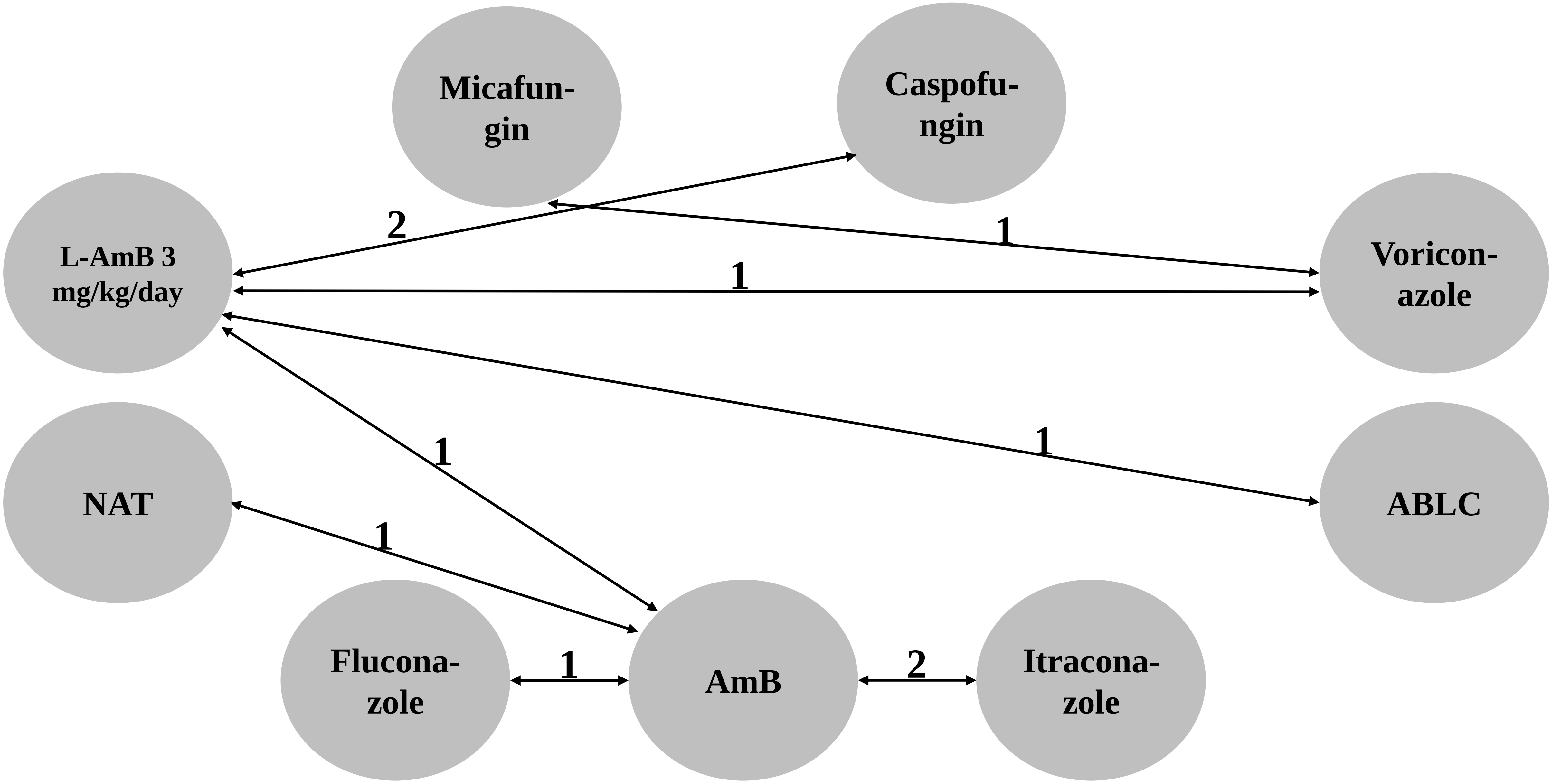

Figure S13. A schematic of the network of evidence for fungal infection-related mortality

ABLC: Amphotericin B lipid complex; AmB: Conventional amphotericin B; L-AmB: Liposomal amphotericin B; NAT: No antifungal treatment

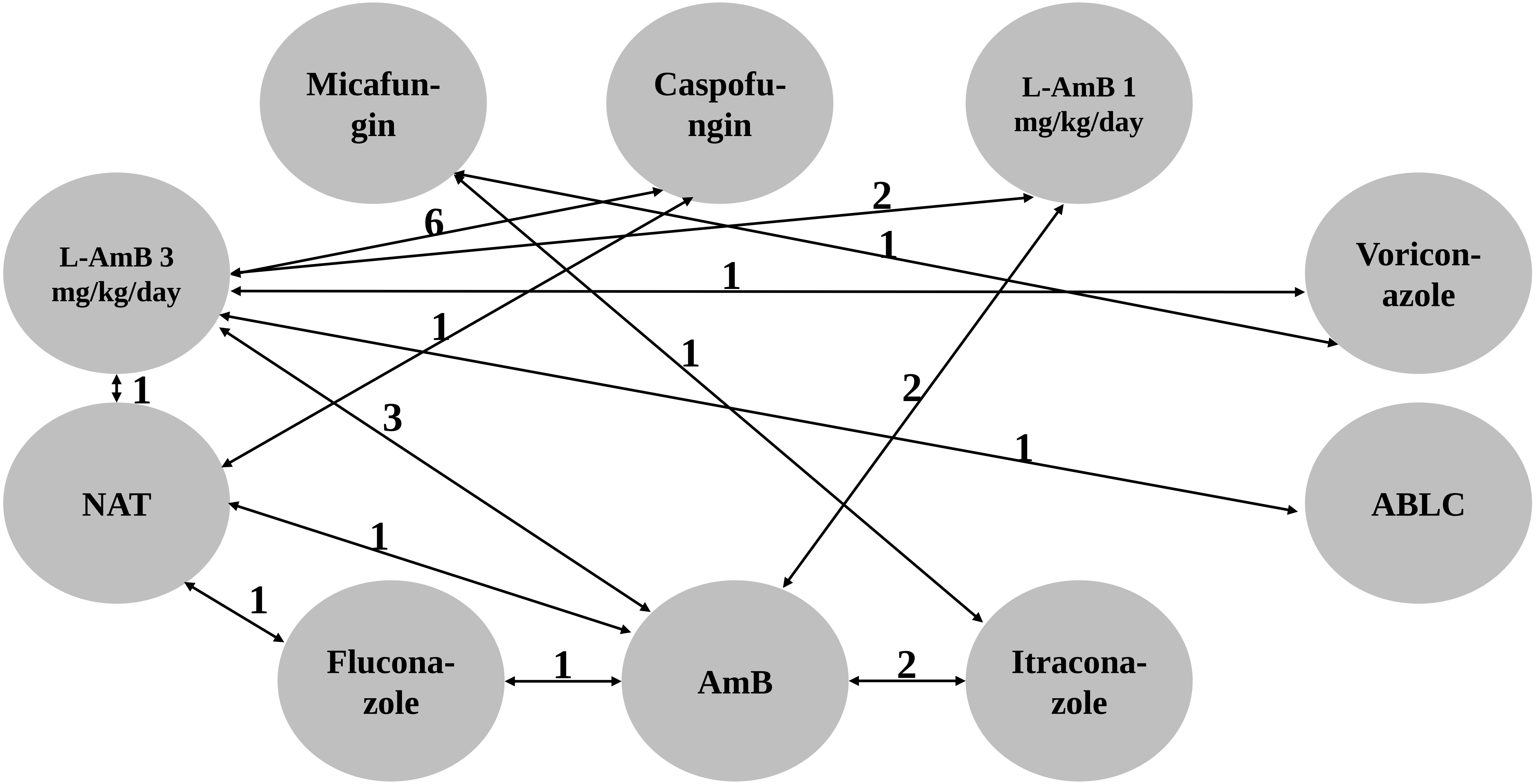

Figure S14. A schematic of the network of evidence for treatment response

ABLC: Amphotericin B lipid complex; AmB: Conventional amphotericin B; L-AmB: Liposomal amphotericin B; NAT: No antifungal treatment

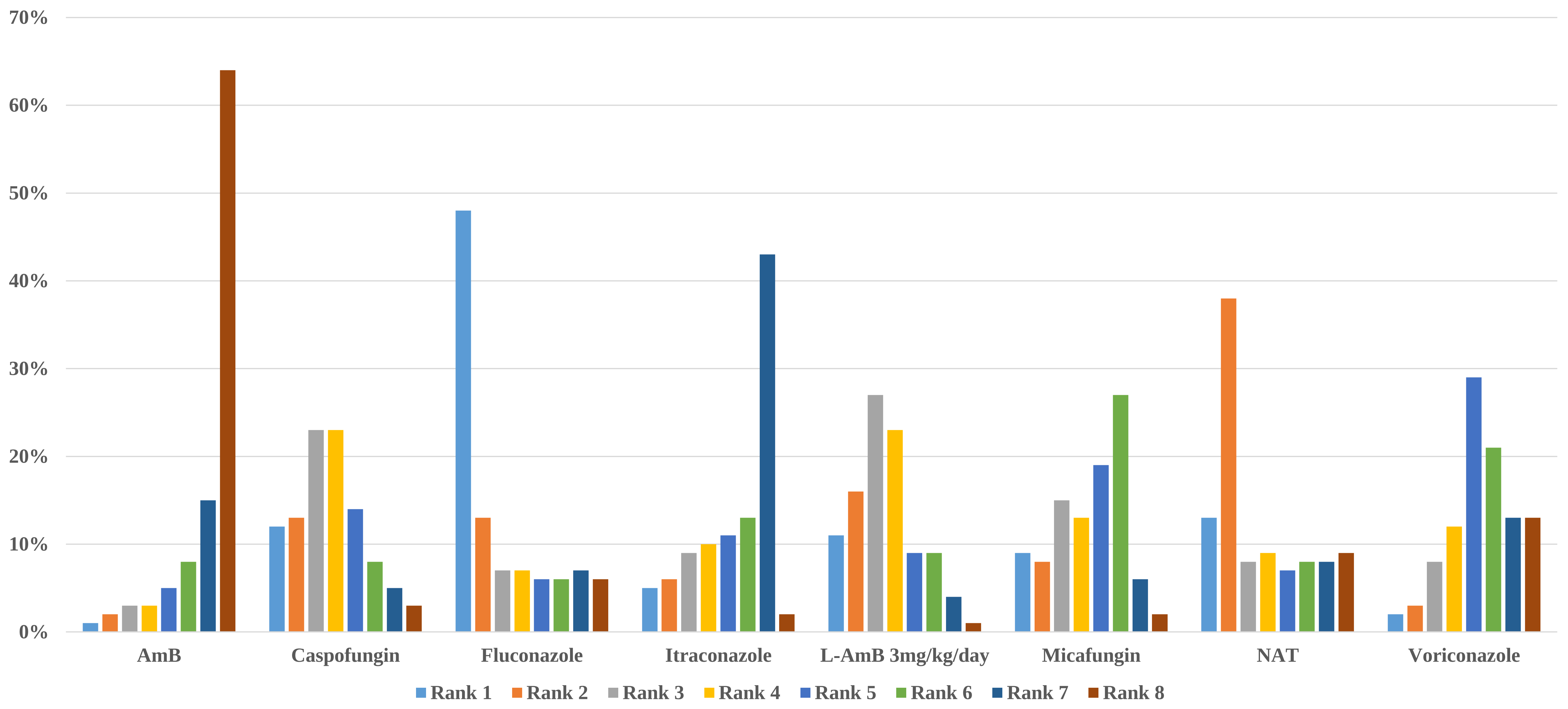

Figure S15. Rank probability plot of antifungals for treatment response in subgroup analysis of non-allogenic haematological stem cell transplantation

AmB: Conventional amphotericin B; L-AmB: Liposomal amphotericin B; NAT: No antifungal treatment

Rank 1 is best, rank 8 is worst.

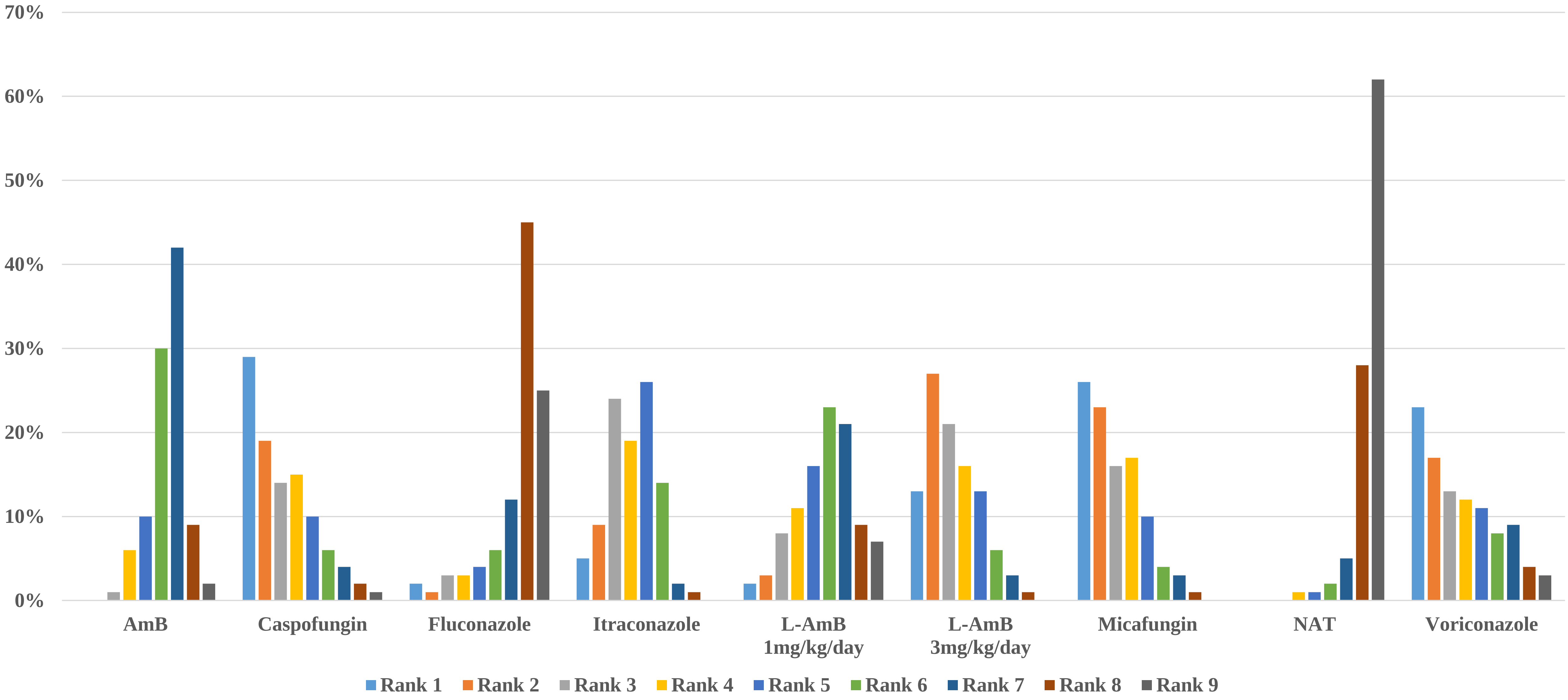

Figure S16. Rank probability plot of antifungals for treatment response in subgroup analysis of adults
AmB: Conventional amphotericin B; L-AmB: Liposomal amphotericin B; NAT: No antifungal treatment
Rank 1 is best, rank 9 is worst.

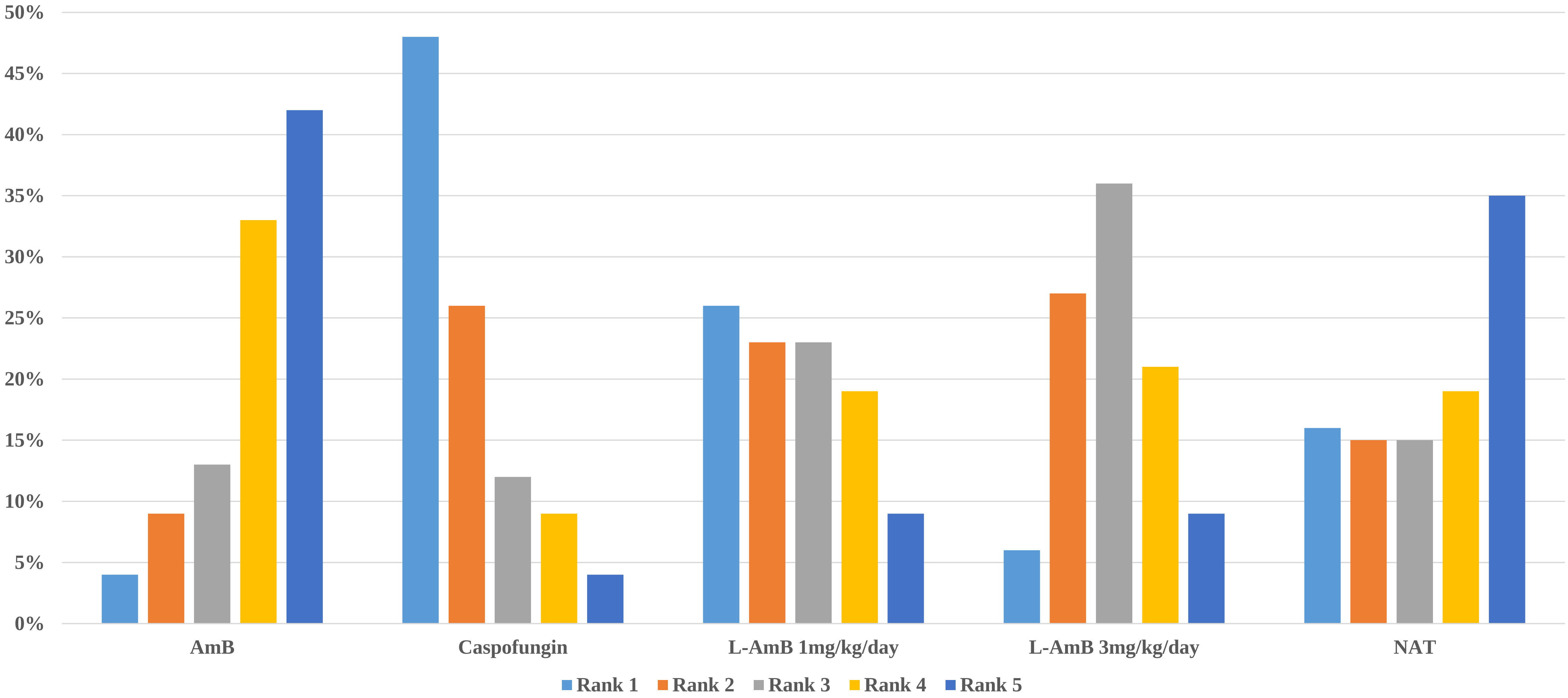

Figure S17. Rank probability plot of antifungals for treatment response in subgroup analysis of pediatrics
AmB: Conventional amphotericin B; L-AmB: Liposomal amphotericin B; NAT: No antifungal therapy
Rank 1 is best, rank 5 is worst.

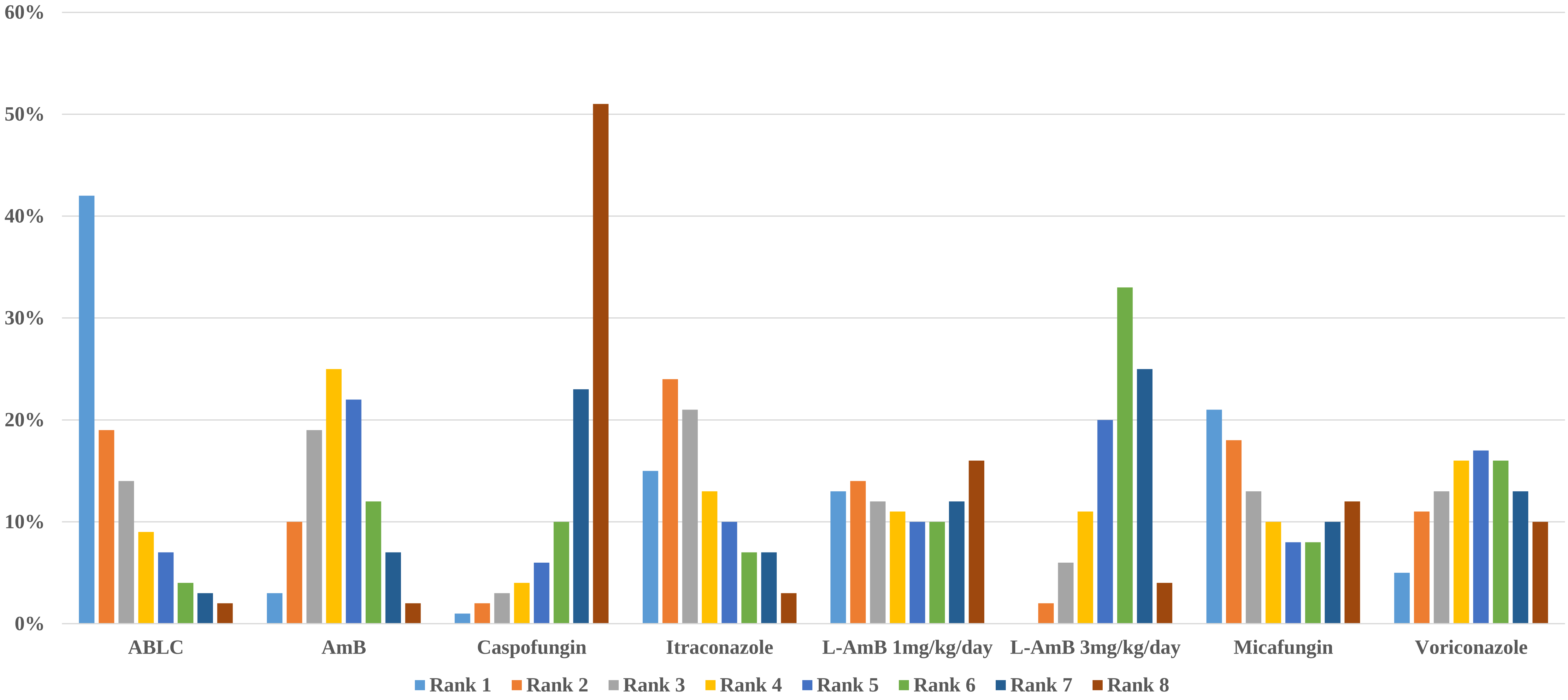

Figure S18. Rank probability plot of antifungals for all-cause mortality in sensitivity analysis
ABLC: Amphotericin B lipid complex; AmB: Conventional amphotericin B; L-AmB: Liposomal amphotericin B
Rank 1 is worst, rank 8 is best.

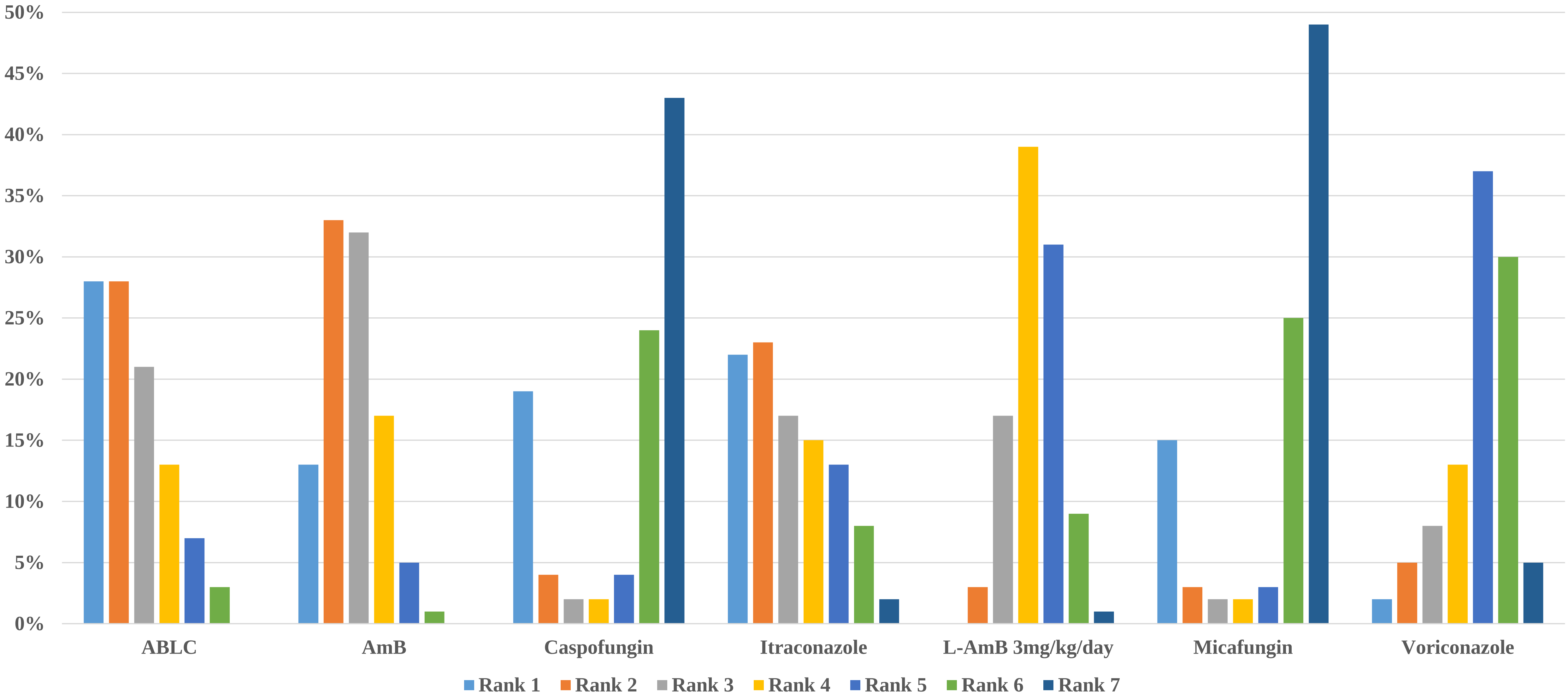

Figure S19. Rank probability plot of antifungals for fungal infection-related mortality in sensitivity analysis
ABLC: Amphotericin B lipid complex; AmB: Conventional amphotericin B; L-AmB: Liposomal amphotericin B
Rank 1 is worst, rank 7 is best.

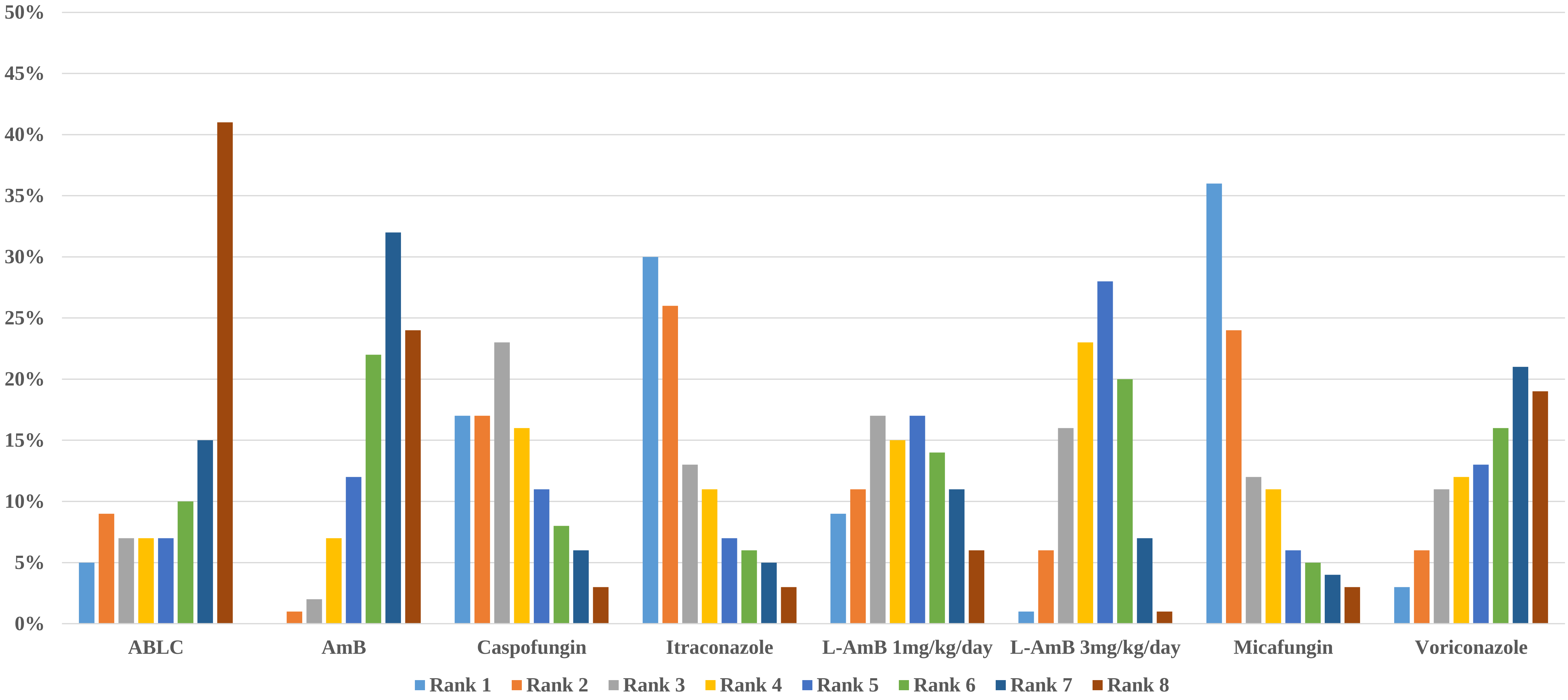

Figure S20. Rank probability plot of antifungals for treatment response in sensitivity analysis
ABLC: Amphotericin B lipid complex; AmB: Conventional amphotericin B; L-AmB: Liposomal amphotericin B
Rank 1 is best, rank 8 is worst.
